# Glycometabolism and lipid metabolism related genes predict the prognosis of endometrial carcinoma and their effects on tumor cells

**DOI:** 10.1186/s12885-024-12327-1

**Published:** 2024-05-08

**Authors:** Xuefen Lin, Jianfeng Zheng, Xintong Cai, Li Liu, Shan Jiang, Qinying Liu, Yang Sun

**Affiliations:** 1 Department of Gynecology, Clinical Oncology School of Fujian Medical University, Fujian Cancer Hospital, No.420, Fuma Road, Jin’an District, Fuzhou City, Fujian Province 350014 P. R. China; 2 Fujian Provincial Key Laboratory of Tumor Biotherapy, Clinical Oncology School of Fujian Medical University, Fujian Cancer Hospital, Fuzhou, 350014 China; 3 Fujian University of Chinese Medicine, Fuzhou, 350014 China

**Keywords:** Endometrial Carcinoma, Carbohydrate metabolism, Lipid metabolism, Prognosis, Hexokinase II

## Abstract

**Background:**

Glycometabolism and lipid metabolism are critical in cancer metabolic reprogramming. The primary aim of this study was to develop a prognostic model incorporating glycometabolism and lipid metabolism-related genes (GLRGs) for accurate prognosis assessment in patients with endometrial carcinoma (EC).

**Methods:**

Data on gene expression and clinical details were obtained from publicly accessible databases. GLRGs were obtained from the Genecards database. Through nonnegative matrix factorization (NMF) clustering, molecular groupings with various GLRG expression patterns were identified. LASSO Cox regression analysis was employed to create a prognostic model. Use rich algorithms such as GSEA, GSVA, xCELL ssGSEA, EPIC,CIBERSORT, MCPcounter, ESTIMATE, TIMER, TIDE, and Oncoppredict to analyze functional pathway characteristics of the forecast signal, immune status, anti-tumor therapy, etc. The expression was assessed using Western blot and quantitative real-time PCR techniques. A total of 113 algorithm combinations were combined to screen out the most significant GLRGs in the signature for in vitro experimental verification, such as colony formation, EdU cell proliferation, wound healing, apoptosis, and Transwell assays.

**Results:**

A total of 714 GLRGs were found, and 227 of them were identified as prognostic-related genes. And ten GLRGs (AUP1, ESR1, ERLIN2, ASS1, OGDH, BCKDHB, SLC16A1, HK2, LPCAT1 and PGR-AS1) were identified to construct the prognostic model of patients with EC. Based on GLRGs, the risk model’s prognosis and independent prognostic value were established. The signature of GLRGs exhibited a robust correlation with the infiltration of immune cells and the sensitivity to drugs. In cytological experiments, we selected HK2 as candidate gene to verify its value in the occurrence and development of EC. Western blot and qRT-PCR revealed that HK2 was substantially expressed in EC cells. According to in vitro experiments, HK2 knockdown can increase EC cell apoptosis while suppressing EC cell migration, invasion, and proliferation.

**Conclusion:**

The GLRGs signature constructed in this study demonstrated significant prognostic value for patients with endometrial carcinoma, thereby providing valuable guidance for treatment decisions.

**Supplementary Information:**

The online version contains supplementary material available at 10.1186/s12885-024-12327-1.

## Introduction

Endometrial carcinoma (EC) is the second most common gynecological cancer in women worldwide [1]. Its prevalence and mortality are both rising annually, which poses a severe threat to women’s health [1]. The increasing incidence of EC is closely related to the increasing incidence of obesity and diabetes worldwide [2]. Studies in epidemiology have demonstrated that obesity constitutes an independent risk factor for EC, with a positive correlation observed between body mass index (BMI) and the incidence of this malignancy [3]. Moreover, metabolic disorders such as diabetes mellitus are related to the incidence and adverse pathological features of EC [4]. The investigation of EC development, particularly the tumor metabolism mechanism, has emerged as a prominent research focus in molecular targeted therapy for EC [5]. Further elucidation of the molecular mechanisms underlying EC in metabolism holds significant clinical therapeutic implications.

The nutrients glucose (carbohydrate) and lipids play crucial roles in the body, closely intertwined with energy storage and supply, and are integral components of cellular metabolic processes [6]. The process of tumor development involves the reprogramming of glycometabolism (carbohydrate metabolism) and lipid metabolism, which is intricately linked to tumor progression, invasion, metastasis, and immune modulation [7, 8]. Glycolysis is the predominant pathway of glycometabolism in the human body. Tumor cells exhibit heightened glucose consumption to rapidly generate sufficient ATP for energy via glycolysis. Even under conditions of adequate oxygen availability, there is a propensity for glucose conversion into lactic acid, known as the Warburg effect [9]. Consequently, the augmented glycolytic activity of malignant tumor cells can induce an acidic microenvironment surrounding them, fostering normal cell death while facilitating tumor cell angiogenesis and invasion [10]. The metabolic characteristics of the glycometabolism pathway and mitochondrial function exhibited significant alterations in EC [11]. Lipids play crucial roles in the composition of cellular membranes, energy metabolism, and synthesis of endocrine hormones [12]. The preservation of cell membrane structure and facilitation of cell signal transduction are critically dependent on cholesterol and phospholipids [13]. Lipids and their metabolic intermediates play a pivotal role in diverse cellular signal transduction pathways implicated in cancer [14]. The dysregulation of lipid metabolism represents a pivotal metabolic alteration in the context of cancer. Lipid metabolism disorder can result in dysregulated expression of various genes and proteins, as well as perturbed cytokine profiles and disrupted signaling pathways [15]. Despite the correlation between the incidence and development of EC with glycometabolism and lipid metabolism [16–18], there remains uncertainty regarding the molecular processes involved and the impact of associated genes on EC patient prognosis. Additionally, current research on abnormal glycometabolism and lipid metabolism in EC patients mainly consists of single-gene laboratory studies, with little exploration into gene clusters associated with these metabolic processes.

The implementation of comprehensive treatment strategies has significantly enhanced the overall prognosis of patients with EC; however, individuals experiencing recurrent and metastatic EC exhibit a dismal survival outlook under the current therapeutic regimen [19]. Moreover, currently there is a lack of robust biomarkers or predictive models that can accurately forecast the survival rate of patients with EC [20]. The conventional methods for tumor histology and morphology classification fail to comprehensively capture the heterogeneity among EC cells and patients, leading to inadequate repeatability [21–23] and significant variations in prognosis even within the same stage of EC [24, 25]. Reclassification of tumors based on various criteria enables the classification of patients, thereby facilitating their management, treatment, and follow-up. The conventional classification of EC is important for diagnosing and treating patients. However, as disease research progresses, the limitations of traditional methods become more evident. Conventional classification heavily depend on clinical or histological features without fully considering genomic characteristics, which can lead to varying outcomes among patients with the identical EC classifications [26–29]. Moreover, there is often an overlap between tissue type and FIGO grade, making it challenging to address tumor heterogeneity and resulting in subjective diagnoses that complicate clinical decision-making. Multiple factors, encompassing genomic and clinical aspects, play a pivotal role in the development and prognosis of endometrial cancer; however, the existing classification system inadequately predicts the survival outcome of patients affected by this malignancy [30].

Due to the limitations of conventional classification and basic experiments, numerous bioinformatics analysis techniques have been extensively employed for the identification and characterization of genes associated with the progression of diverse cancer types. Our previous studies have focused on the domain of biomarker screening and bioinformatics analysis employing high-throughput sequencing technology from public databases [31, 32]. The emergence of pathogenic abnormalities stems from intricate network relationships among genes [33]. Therefore, in our study, we identified ten glycometabolism and lipid metabolism-related genes (GLRGs) from the Genecards database to construct a reliable prognostic signature for predicting overall survival (OS) and treatment strategies. Our data demonstrated a significant correlation between the GLRG-related prognostic signature and immune features, tumor mutational burden (TMB), as well as chemosensitivity. Furthermore, we successfully validated the impact of this representative gene model on EC cells in vitro.

## Material and methods

### Data collection for EC

We downloaded the processed data containing RNA sequences and clinical information (such as prognostic information) from The Cancer Genome Atlas (TCGA) (https://portal.gdc.cancer.gov/, accessed on 5 July 2023) [34] and the Genotype-Tissue Expression (GTEx) project (https://www.gtexportal.org/home/, accessed on 5 July 2023) [35]. The primary EC patients with insufficient clinical data and follow-up information were excluded, resulting in the inclusion of 545 primary EC patients and 78 samples from healthy individuals. According to gene annotation information in GENCODE, the Ensemble Gene was transformed into a Gene Symbol [36]. The Genecards database (https://www.genecards.org/, accessed on 6 July 2023) was utilized to retrieve a total of 714 GLRGs (Supplementary Table S1) [37]. Using the Maftools package, the somatic mutations of mRNAs were created using the Mutation Annotation Format (MAF) [38]. We obtained EC, or normal control endometrial tissues, following the approval of the ethics committee at Fujian Cancer Hospital.

### Differential and prognostic analysis of GLRGs

We screened differentially expressed GLRGs using limma package (*P*<0.05 and |logFC|>1) [39]. The volcano plot for differentially expressed GLRGs was generated using the ggplot2 package. The Survminer package [40] was utilized to identify the optimal cut-point and select prognostic GLRGs based on the expression level of differential GLRGs, survival time, and state.

### Establishment of the GLRG-related cluster and signature

Based on the differentially expressed GLRGs with prognostic value, EC patients were grouped using the non-negative matrix factorization (NMF) clustering algorithm [41]. By leveraging expression levels of individual GLRGs and employing Least Absolute Shrinkage and Selection Operator (LASSO) regression prognostic coefficient [42], we have developed a risk-score (RS) model as follows: Risk Score =∑β_gene_×Exp_gene_. In the RS formula, β_gene_ is the LASSO regression coefficient of the GLRG, and Exp_gene_ signifies the expression level of the GLRG. The RS of each EC patient was calculated, and the median RS was used as the critical value to further divide the EC patients into high-risk group and low-risk group (high-risk group: RS≥median; low-risk group: RS < median). Considering the sample size and referring to previous literature [43, 44], the total samples (Total Set, *n*=545) were randomly divided into a Train Set (*n*=273) and a Test Set (*n*=272) in a one-to-one ratio using the random sampling function in the R programming language to minimize information leakage and enhance model performance evaluation accuracy. Initially, the risk scores for patients in the Training Set were computed using the aforementioned formula. Subsequently, we employed the same methodology to calculate the risk scores of both the Testing Set and Total Set for validation purposes. The Kaplan-Meier curves were utilized to evaluate the survival outcomes of different risk groups. To assess the GLRG-related signature’s prediction power, we created ROC curves using the timeROC program. The autonomous prognostic relevance of the linked components, such as RS and clinical characteristics, was confirmed by the Univariate and Multivariate Cox regression models. The calibration curve was used to confirm the construction of the visual nomogram and assess the risk model’s accuracy for use as a stand-alone prognostic factor [45]. Besides, principal component analysis (PCA) [46] and t-distributed stochastic neighbor embedding (t-SNE) [47] were applied to test the effectiveness of the risk scores in differentiating EC patients.

### Enrichment analysis of candidate genes

Gene Ontology (GO) and Kyoto Encyclopedia of Genes and Genomes (KEGG) enrichment analyses were carried out utilizing the ClusterProfler package [48]. Gene Set Variation Analysis (GSVA) was conducted utilizing the GSVA package [49]. The Gene Set Enrichment Analysis (GSEA) pathway enrichment analysis was conducted to compare the two different groups [50].

### Evaluation of immune activity and therapy for the two GLRG-related risk groups

The immunological microenvironment has a significant role in the origin and evolution of EC. An evaluation of the immune-infiltrating cell abundance was conducted using the IOBR algorithm [51]. We utilized the TIDE algorithm to predict potential responses to immune checkpoint blockade (ICB) [52]. We employed the web platforms GenePattern and Submap to conduct a comparative analysis of immunotherapy disparities between the two risk groups [53]. The oncoPredict package [54] was utilized to evaluate the IC50 levels of chemotherapy drugs for patients with EC.

### Screening significant GLRGs in EC using machine learning-dependent integrative approaches

The most significant GLRGs among the GLRGs in the GLRG-related signature were further screened out by integrating 12 machine learning (ML) algorithms and combining 113 algorithm combinations [55] to authenticate GLRGs with high accuracy and stability between EC and normal samples. As previously mentioned, considering the sample size and referring to previous literature [43, 44], the total samples (All Set) were randomly and equally partitioned into a Train Set and a Test Set. The same Train Set was utilized to construct signatures among the 113 algorithms, and subsequently, the validation of these signatures was performed based on the calculation results obtained from both the same Test Set and All Set. The frequency of GLRGs observed in the 113 algorithm combinations was computed, and subsequently, the GLRG with the highest occurrence rate was chosen for subsequent wet experiments.

### Cell lines and cell culture

The endometrial epithelial cells (EECs) were preserved in our laboratory. The extensively employed EC cells, HEC-1A and Ishikawa, were obtained from the Meisen Chinese Tissue Culture Collections (Zhejiang, China) and preserved in our laboratory. McCoy’s 5A medium (Shanghai BasalMedia Technologies Co., Ltd., Shanghai, China) mixed with 10% fetal bovine serum (FBS) was used to cultivate HEC-1A cells. RPMI 1640 medium (Shanghai BasalMedia Technologies Co., Ltd.) containing 10% FBS was used to cultivate Ishikawa cells and EECs. The cell culture medium was supplemented with streptomycin at a concentration of 100 g/mL and penicillin at a concentration of 100 U/mL (Beyotime, Beijing, China). Cells were grown at 37°C in a 5% CO_2_ environment.

### Quantitative real-time PCR

The extraction of total RNA was performed using TRNzol Universal Reagent (Tiangen Biotech, Beijing, China). Reverse transcription was performed using FastKing gDNA Dispelling RT SuperMix (Tiangen Biotech, Beijing, China). The reaction was conducted at a temperature of 42°C for a duration of 15 minutes to facilitate genome removal and reverse transcription, followed by an enzyme inactivation step at 95°C for 3 minutes. SuperReal PreMix Plus (Tiangen Biotech, Beijing, China) was utilized for quantitative real-time PCR (qRT-PCR). The initial denaturation step was performed at 95°C for 15 minutes, followed by a total of 40 cycles consisting of denaturation at 95°C for 10 seconds and annealing/extension at 60°C for 32 seconds in the RT-qPCR cycling conditions. The melt curve stage was programmed as follows: 95°C for 15 seconds, 60°C for 60 seconds, 95°C for 15 seconds, and 60°C for 10 seconds in accordance with the instructions provided in the kit. The primer sequences utilized in this study can be found in Supplementary Table S2. The β-actin PCR product, with the size of 248 bp, exhibited a homogeneous melting temperature of approximately 87.5 °C. The HK2 PCR product, with the size of 87 bp, displayed the homogeneous melting temperature around 83 °C. The expression levels were calculated using the 2^-ΔΔCt^ formula.

### Cell transfection for EC cells

Observing the guidelines provided by the manufacturer, small interfering RNAs (siRNA) targeting HK2 and negative control RNAs (GenePharma, Shanghai, China) were employed for silencing HK2 expression using a GP-transfect-mate (GenePharma, Shanghai, China) transfection reagent. The specific sequences for siRNA can be found in Supplementary Table S3.

### Western blot assays

The total proteins were extracted using RIPA buffer supplemented with a proteinase inhibitor cocktail (Beyotime, Beijing, China). Utilizing the BCA assay kit (Beyotime, Beijing, China), the protein content was determined. Polyacrylamide gel electrophoresis (SDS-PAGE) with the addition of sodium dodecyl sulfate was employed to separate the proteins, followed by their transfer onto the polyvinylidene fluoride membranes made from PVDF. After an hour of room temperature blocking with blocking solution, the membranes were incubated for an entire night at 4°C with primary antibodies targeting HK2 (dilution 1:5000, Cat No. 66974-1-Ig, Proteintech, China) and β-Actin (dilution 1:5000, Cat No. 81115-1-RR, Proteintech, China). Following a wash, the membrane was left at room-temperature for an hour to be incubated with the secondary antibody (dilution 1:10000, ZB-2306, ZSGB-BIO, China). An improved chemiluminescent substrate was used to identify protein bands.

### CCK-8 assays for EC cells

After transfection, the transfected cells were cultured for four days in 96-well plates with a seeding density of 2000 cells/well and 100 μl of complete growth medium. The Counting Kit-8 (APExBIO, Houston, TX, USA) was utilized to measure the optical density (OD) at 450 nm using a computerized microplate reader at time points of 0-hour, 24-hour, 48-hour, 72-hour, and 96-hour in accordance with instructions. The groups were equipped with three multiple wells each and each trial was repeated in triplicate.

### Scratch wound-healing assays for EC cells

Upon reaching approximately 100% confluency on the 6-well plates, the EC cells were gently scraped with a 200µl pipette tip, and the suspended cells were subsequently removed with PBS. Afterwards, the EC cells were cultured in a serum-free medium at 37°C with 5% CO_2_. Subsequently, three non-overlapping views of each well were randomly captured at both the 0-hour and 48-hour time points. The pictures were imported into Image J, an image processing program, and examined. The following formula calculates the rate of wound healing: wound healing rate = (A_0h_–A_48h_)/A_0h_×100%, where A_0h_ is the initial wound area and A_48h_ is the wound area left at the end of the healing process.

### Transwell assays for EC cells

The Transwell assays were conducted using chambers (pore size, 8 µm) equipped with polycarbonate filters. In the upper compartment, 2×10^5^ EC cells were put in 300 μL of serum-free medium, while the lower chamber contained 500 μL of McCoy’s 5A media supplemented with 30% FBS. For the invasion experiment, Matrigel was applied to the chamber. The EC cells were cultured for 48 hours at a temperature of 37°C in an environment containing 5% CO_2_. The cells that were unable to pass through the transwell were removed using cotton swabs. Following a ten-minute fixation in 4% paraformaldehyde, the cells that migrated to the lower chamber were subsequently stained with 0.1% crystal violet for five minutes. Finally, cell quantification on the lower surface was calculated using a microscope.

### Colony formation assays for EC cells

In 6-well culture plates, 500 cells were added to each well for a 14-day inoculation period. When discernible clones appeared, the supernatant was removed. After being cleaned twice with PBS, it was fixed for 15-20 minutes by adding 1 mL of 4% paraformaldehyde fixative. After removing the supernatant, the sample was subjected to two rounds of PBS washing. Subsequently, 1 mL of crystal violet staining solution was added and incubated for a duration of 15 to 20 minutes. The culture plates were carefully rinsed with tap water, allowed to dry naturally, and then photographed, with the number of clones that could be seen being tallied.

### Apoptosis assays for EC cells

The apoptosis assays were carried out according to the instructions of the manufacturer (MedChemExpress, NJ, USA). Following transfection, 195 μl of binding buffer was used to resuspend the cells after they had been cleaned with PBS and digested with trypsin without EDTA. Subsequently, the cells were stained with 10 μl of Annexin V-FITC and 5 μl of propidium iodide (PI), followed by a dark incubation at room temperature for 15 minutes. The apoptosis of cells was quantified using a flow cytometer (BD Biosciences, New York, USA).

### EdU cell proliferation assays for EC cells

HK2 knock-down and the matching control EC cells were seeded at a density of 2×10^5^ cells/well in 24-well plates and cultivated for 24 hours. The assessment of cell proliferation was conducted using the EdU analysis kit (APExBIO, Houston, TX, USA) in accordance with the provided instructions.

### Statistical analysis of this study

The statistical analysis was conducted using the R package (v 4.0.2) or GraphPad Prism (v 9.0). The assay was performed in triplicate and the results were presented as Mean ± SD. The normality of data distribution was analyzed by normality tests (D’Agostino-Pearson omnibus, Anderson-Darling, Shapiro-Wilk and Kolmogorov-Smirnov) provided by GraphPad Prism. The survival outcome between two subgroups was compared using Kaplan-Meier curves and the log-rank test. The Spearman correlation coefficient was employed to ascertain the association between the two variables. The two-tailed Student’s t-test was employed for comparing data between two groups, while the one-way ANOVA test was utilized for analyzing data from more than two groups. Additionally, specialized analyses were detailed in the corresponding section. The threshold for determining a statistically significant difference was set at *P*<0.05, adhering to the conventional criterion.

## Results

### Expression and functional analysis of GLRGs

**Fig. 1:**
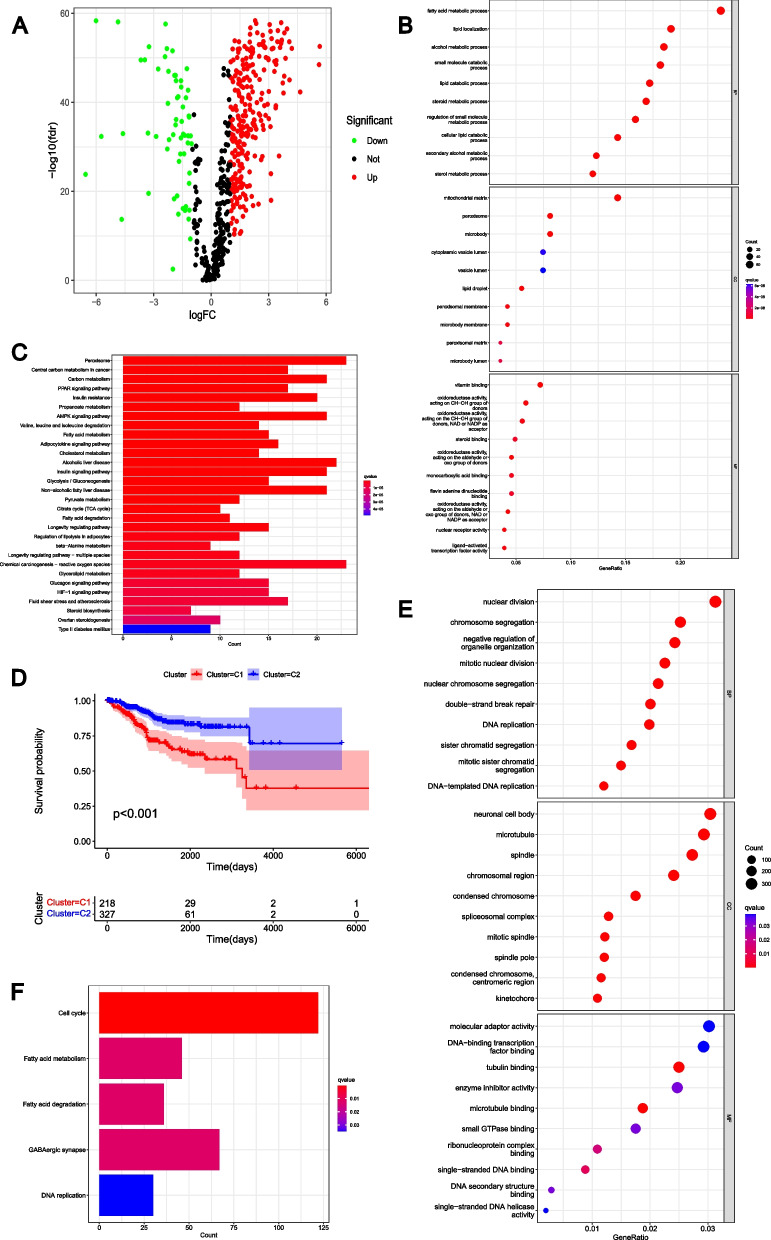
Expression and functional analysis of GLRGs. **A** Volcano map of differentially expressed GLRGs in EC. The red dots represented highly expressed genes, and the green dots represented low-expressed genes. **B** The GO functional enrichment analysis of the differential GLRGs. The size of the dots indicates the number of genes attributed to the corresponding category. The color of the dots represented the q value. **C** KEGG pathway analysis of the differentially expressed GLRGs. The color of the bars represented the q value. **D** The survival curves of the molecular subtypes. The red curve represented Cluster I and the blue curve represented Cluster II. **E** The GO functional enrichment analysis of the differentially expressed genes between Cluster I and Cluster II. The size of the dots indicates the number of genes attributed to the corresponding category. The color of the dots represented the q value. **F** KEGG pathway analysis of the differentially expressed genes between Cluster I and Cluster II. The color of the bars represented the *q* value

The analysis revealed a total of 317 differentially expressed GLRGs in EC (Fig. 1A), including 257 highly expressed GLRGs and 60 lowly expressed GLRGs in EC (Supplementary Table S4). The functions of the 317 GLRGs were analyzed using GO (Fig. 1B) and KEGG analyses (Fig. 1C). The 317 differential GLRGs of EC demonstrated significant enrichment across multiple terms, such as fatty acid metabolic process, lipid localization, lipid catabolic process, sterol metabolic process and so on (Fig. 1B). Additionally, KEGG analysis revealed significant enrichment of the 317 differentially expressed genes related to metabolism in EC within various metabolic pathways, including Central carbon metabolism in cancer, Propanoate metabolism, Glycolysis/Gluconeogenesis, Fatty acid metabolism, Pyruvate metabolism, Carbon metabolism, Citrate cycle (TCA cycle) and so on (Fig. 1C).

### Molecular clusters based on GLRGs

The NMF method was used to establish molecular subtypes for EC. According to cophenetic, dispersion, and silhouette, the selection of two clusters was the ideal number (Supplementary Figure S1A-B). The patients with EC were categorized into GLRG-related Cluster 1 (C1) and Cluster 2 (C2). The prognosis of the two GLRG-related clusters was further examined. The prognosis of EC patients in C2 was observed to be more favorable, whereas EC patients in C1 exhibited a poorer prognosis (Fig. 1D). We analyzed differentially expressed genes (DEGs) across C1 and C2 to look into the biological role of the GLRG-related clusters. Based on GO terms (Fig. 1E) and KEGG pathways (Fig. 1F), we identified that these DEGs were associated with crucial biological processes, including DNA replication, DNA−templated DNA replication, Cell cycle, Fatty acid metabolism, GABAergic synapse, and so on.

### Construction and validation of the risk model based on GLRGs

**Fig. 2:**
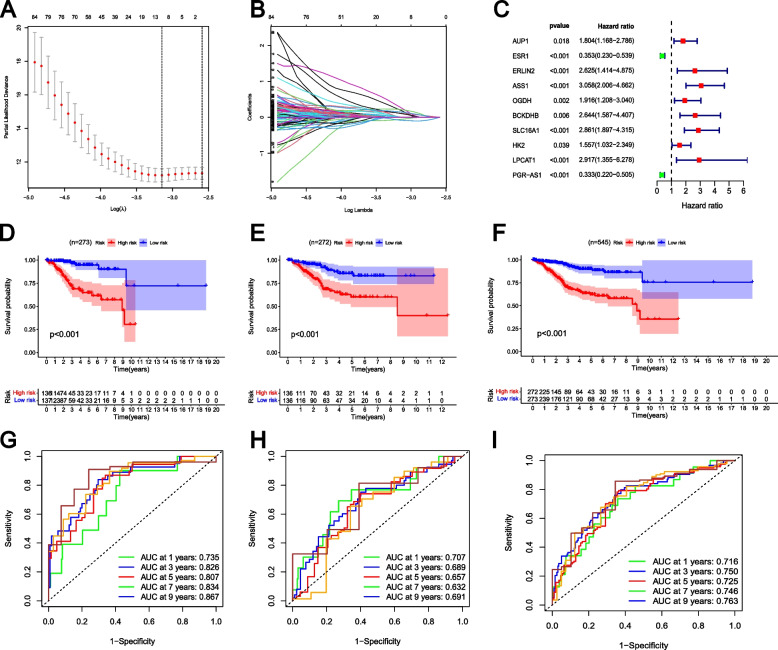
Construction and validation of the risk model based on GLRGs. **A** Cross-validation for tuning the parameter selection in the LASSO regression. **B** LASSO regression of the ten prognostic GLRGs. **C** The ten prognostic GLRGs extracted by Univariate Cox regression analysis were shown in the forest map. **D-F** The K-M survival curves of Train Set (**D**), Test Set (**E**) and Total Set (**F**) based on the GLRG-related risk model. The red curve represented the high-risk group, and the blue curve represented the low-risk group. **G-I** Time-dependent ROC curve analysis of Train Tet (**G**), Test Set (**H**) and Total Set (**I**)

The 227 prognostic GLRGs **(**Supplementary Table S5) utilized for conducting LASSO Cox analysis, thereby constructing the prognostic GLRG-related signature. The λ selection diagram was shown in Fig. 2A-B. A total of ten GLRGs (AUP1, ESR1, ERLIN2, ASS1, OGDH, BCKDHB, SLC16A1, HK2, LPCAT1, and PGR-AS1) were selected for the construction of the risk model (Fig. 2C). Given the pivotal role of protein interactions in determining protein function, we used the STRING database (https://cn.string-db.org/, accessed on 24 March 2024) to analyze in detail the interrelationship and nature of the ten GLRGs in the risk model (Supplementary Figure S2A). In the STRING database, the analyzed gene-encoded proteins formed a closely interconnected protein-protein interaction network comprising 5 edges and 9 nodes (PPI enrichment *p*-value:0.00349; average node degree:1.11, average local clustering coefficient:0.444). The protein-protein interactions were graphically depicted as edges, with the strength of these interactions indicated by the assigned weights on each edge. In this particular context, edges represented protein-protein associations that signify precise and significant connections. In other words, although physical attachment is not necessarily implied, these proteins collaborate to facilitate a shared biological function. Our analysis revealed a significant enrichment of genes encoded by the selected participants in a specific Cellular Component, as well as two distinct Subcellular localizations and two Protein Domains.

Survival analysis showed that two GLRGs (ESR1 and PGR-AS1) were protective factors for HR<1, and eight GLRGs (AUP1, ERLIN2, ASS1, OGDH, BCKDHB, SLC16A1, HK2, and LPCAT1) were risk factors for HR>1 (Fig. 2C). The EC patients in the Train (Fig. 2D), Test (Fig. 2E), and Total (Fig. 2F) sets were split into high-risk group and low-risk group based on the median risk score. The correlation between the risk score and survival prognosis was evaluated by generating Kaplan-Meier curves. The distribution of risk scores in the two risk groups for the Train (Supplementary Figure S 2B), Test (Supplementary Figure S 2C), and Total ( Supplementary Figure S 2D) sets were illustrated in Supplementary Figure S 2B-D. The overall survival (OS) of high-risk EC patients was significantly lower than that of the low-risk group in the Train (Fig. 2D), Test (Fig. 2E), and Total sets (Fig. 2F). The accuracy of the risk model in predicting survival status was further confirmed by consistent findings regarding progression-free survival (PFS, Supplementary Figure S 3). The time-varying ROC curve demonstrated the robustness of the prognostic GLRG-related signature in predicting 1-year, 3-year, 5-year, 7-year, and 9-year survival for EC in the Train (Fig. 2G), Test (Fig. 2H), and Total (Fig. 2I) sets.

### Comparison of clinical features and GLRG-related signature

**Fig. 3:**
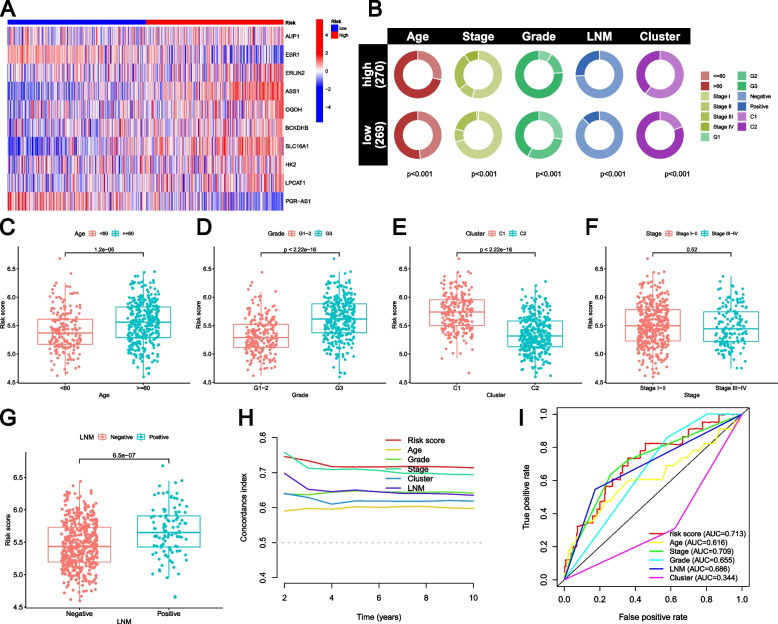
Comparison of clinical features and GLRG-related signature. **A** Heatmap of the expression levels of the ten GLRGs contained in the GLRG-related signature. **B** The pie chart showing the proportion of patients in the two risk groups for each clinical feature. **C-F** Differences in risk scores among the clinical features, including Age (**C**), Grade (**D**), Cluster (**E**), Stage (**F**), and LNM (**G**). **H** Concordance Index curves of risk score and clinical features. **I** The AUC of risk score and clinical characteristics

The expression levels of eight GLRGs (AUP1, ERLIN2, ASS1, OGDH, BCKDHB, SLC16A1, HK2, and LPCAT1) were higher in EC patients with high RSs, while the expression levels of two GLRGs (ESR1 and PGR−AS1) were observed to be downregulated in EC patients with low-risk scores (Fig. 3A; Supplementary Figure S4). EC patients with high-risk scores exhibited advanced age, higher stages and grades, as well as a higher positive rate of lymph node metastasis (LNM) overall (Fig. 3B). The majority of patients with low-risk scores were classified as Cluster 2 (Fig. 3B). Furthermore, the older patients (Fig. 3C), those with higher Grade (Fig. 3D), Cluster 1 (Fig. 3E), and LNM-positive patients (Fig. 3G) exhibited elevated risk scores. However, no statistically significant difference was found in terms of Stage (Fig. 3F). According to the curve, the C-index (Fig. 3H) and AUC (Fig. 3I) of the risk score exhibited superior performance compared to other clinical features, indicating stronger predictive power and higher confidence in the GLRG-related signature.

**Fig. 4:**
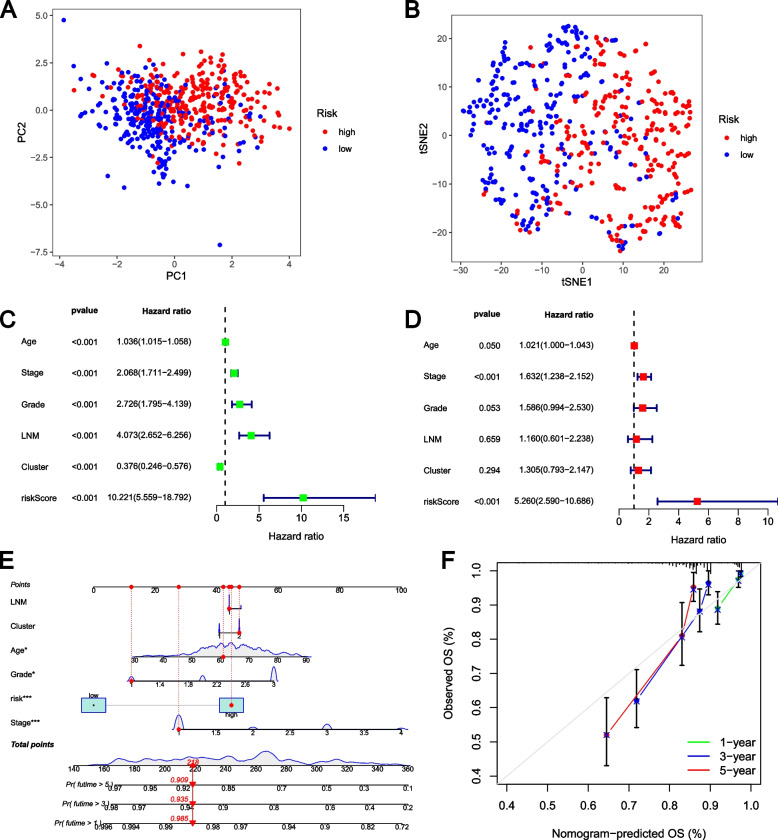
Clinical value of risk score by independent prognostic analysis. **A** PCA analysis of the two risk groups. **B** t-SNE analysis of the two risk groups. **C** The Univariate analysis of risk model and clinical features. **D** The Multivariate analysis of risk model and clinical features. **E** The Nomogram model based on risk model and clinical features. **F** The calibration curve of the risk model

Additionally, the GLRG-related signature significantly distinguished patients with EC, as demonstrated by PCA (Fig. 4A) and t-SNE (Fig. 4B) analyses. Furthermore, the Univariate (Fig. 4C) and Multivariate (Fig. 4D) Cox regression analyses showed that the risk model independently influences the prognosis of patients with EC. The nomogram we constructed can be visually presented in Fig. 4E. The calibration curve was used to evaluate the accuracy of the nomogram in predicting overall survival (OS), and the closer to the ideal matching line or gray line, the better the matching effect (Fig. 4F).

**Fig. 5:**
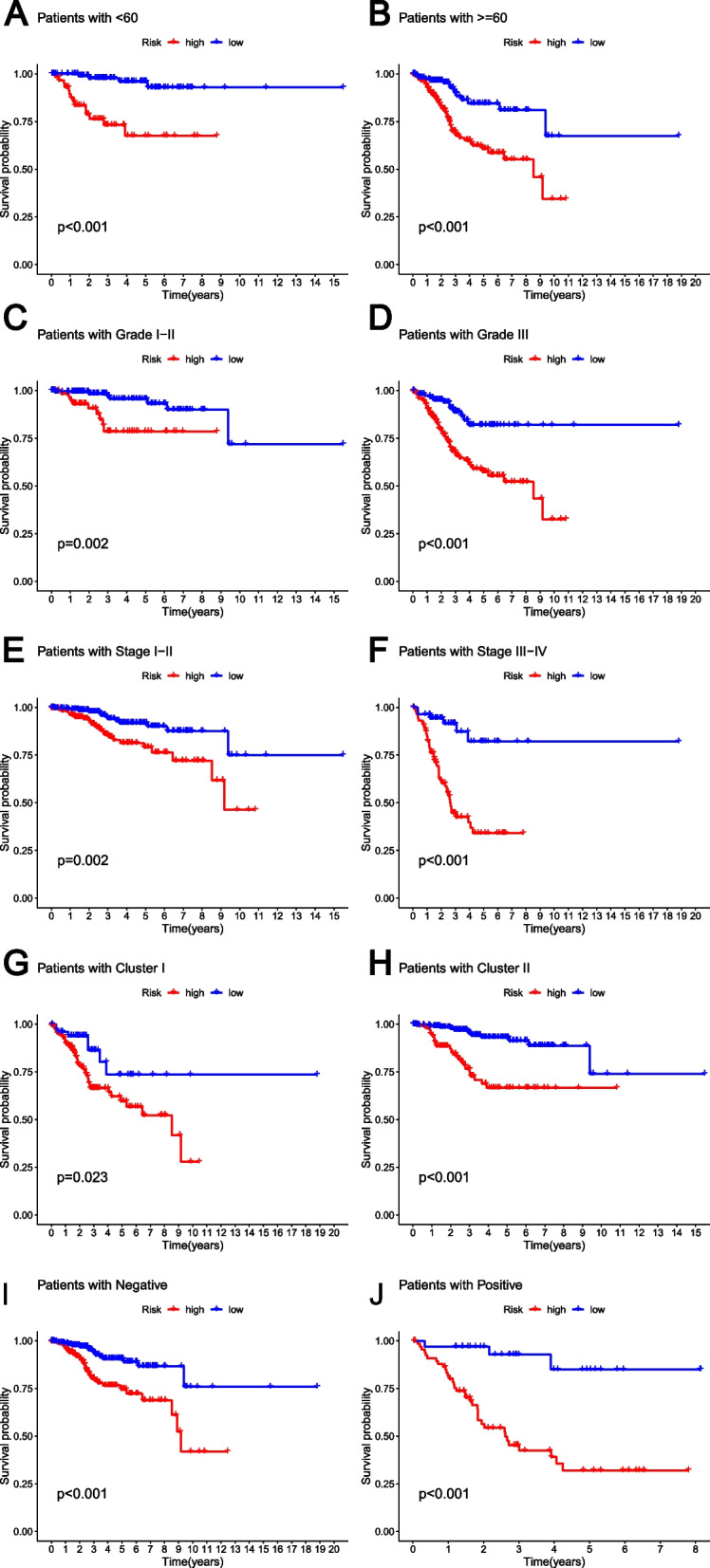
The clinicopathological stratified analysis exploring the prognostic capacity of the GLRG-related signature. **A-H** The K-M survival curves of EC patients in the risk groups considering clinicopathology subgroups, including Age<60 (**A**), Age>=60 (**B**), Grade I-II (**C**), Grade III (**D**), Stage I-II (**E**), Stage III-IV (**F**), Cluster I (**G**), Cluster II (**H**), LNM Negative (**I**), and LNM Positive (**J**)

We conducted clinicopathological stratified analysis to investigate the prognostic potential of the GLRG-related signature with respect to Age, Grade, Stage, GLRG-related Cluster and LNM. Our findings demonstrated a significantly lower overall survival rate for high-risk patients compared to low-risk patients in various subgroups of clinical pathologies, including Age<60 (Fig. 5A), Age>=60 (Fig. 5B), Grade I-II (Fig. 5C), Grade III (Fig. 5D), Stage I-II (Fig. 5E), Stage III-IV (Fig. 5F), Cluster I (Fig. 5G), Cluster II (Fig. 5H), LNM Negative (Fig. 5I) and LNM Positive (Fig. 5J).

### Functional pathways of the risk groups based on GLRGs

**Fig. 6:**
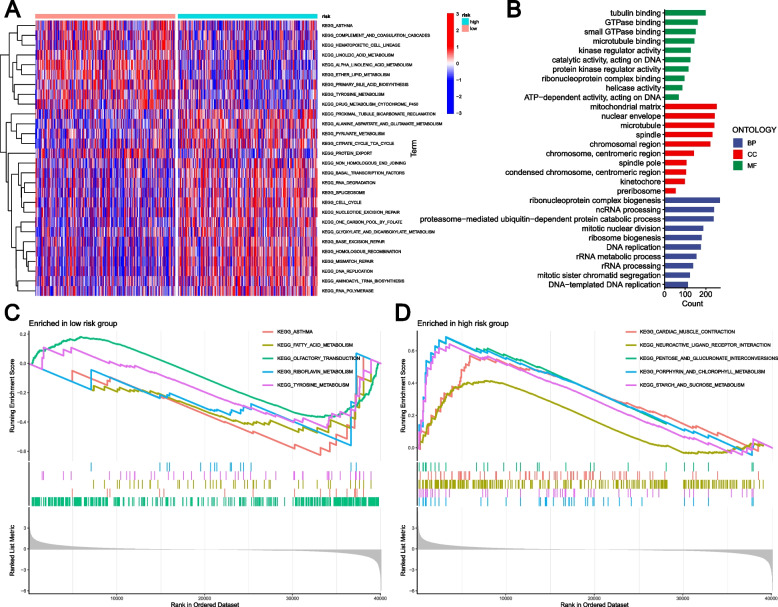
Functional pathways of the risk groups based on GLRGs. **A** The GSVA analysis of two risk subgroups. **B** GO analysis between high-risk group and low-risk group. **C** The top five significant enrichment pathways in the low-risk group by GSEA enrichment analysis. **D** The top five significant enrichment pathways in the high-risk group by GSEA enrichment analysis

The GSVA algorithm-based functional pathway enrichment analysis revealed 28 pathways with significant variations between the two risk groupings (Fig. 6A). The low-risk group exhibited enrichment in several pathways related to metabolism, such as Linoleic Acid Metabolism, Ether Lipid Metabolism, Primary Bile Acid Biosynthesis, Tyrosine Metabolism, Alpha Linolenic Acid Metabolism, and Drug Metabolism Cytochrome P450 (Fig. 6A). Additionally, a number of pathways linked to metabolism were enhanced in the high-risk group, such as Pyruvate Metabolism, Alanine Aspartate and Glutamatemetabolism, and Glyoxylate And Dicarboxylate Metabolism (Fig. 6A). The differentially expressed genes (DEGs) of the risk groups were investigated. The results of GO analysis revealed a significant enrichment of these DEGs in several crucial biological processes (Fig. 6B). The low-risk group had 45 highly enriched pathways, while the high-risk group had 140 significantly enriched pathways based on the GSEA analysis. Notably, our analysis revealed a significant association between low-risk scores and several pivotal metabolic processes, including Fatty Acid Metabolism, Riboflavin Metabolism, and Tyrosine Metabolism (Fig. 6C). Additionally, the high-risk scores were associated with Porphyrin And Chlorophyll Metabolism and Starch and Sucrose Metabolism (Fig. 6D).

### Evaluation of mutations between the two GLRG-related risk groups

**Fig. 7:**
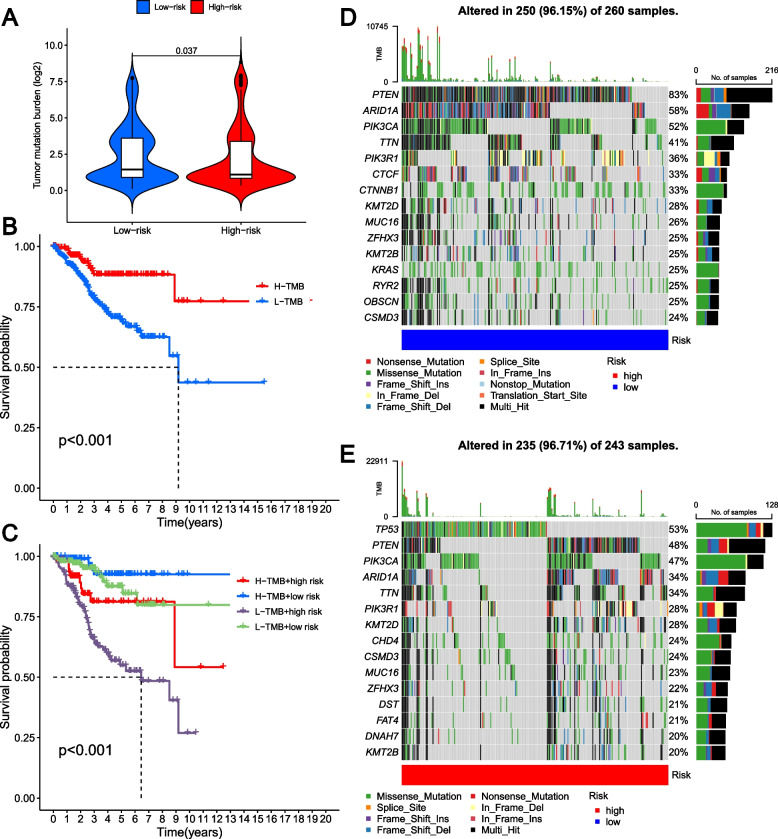
Evaluation of mutation between the two GLRG-related risk groups. **A** The level of TMB between high-risk group and low-risk group. **B** Survival analysis of the different groups stratified by TMB. **C** Survival analysis of distinct groups stratified by both TMB and signature. **D-E** The waterfall plot of somatic mutation features established with low (**D**) and high (**E**) risk scores

The analysis of mutational data from the TCGA-UCEC cohort revealed a higher tumor mutation burden (TMB) in the low-risk group compared to the high-risk group, as shown in Fig. 7A. Based on the optimal cut-point determined for TMB levels, EC patients were categorized into two groups (low-TMB or high-TMB). We found a more favorable prognosis for EC patients in the high-TMB group (Fig. 7B). Our study also revealed that patients with EC had an increased risk when presenting both low TMB levels and high risk scores (Fig. 7C). The waterfall diagram visually illustrates the top 15 mutated genes (Fig. 7D-E), highlighting a molecular disparity between risk groups. The patients with low risk scores exhibited the highest mutation rate of PTEN (Fig. 7D), while the patients with high risk scores demonstrated the highest mutation rate of TP53 (Fig. 7E).

### Evaluation of immune activity and immunotherapy for the two GLRG-related risk groups

**Fig. 8:**
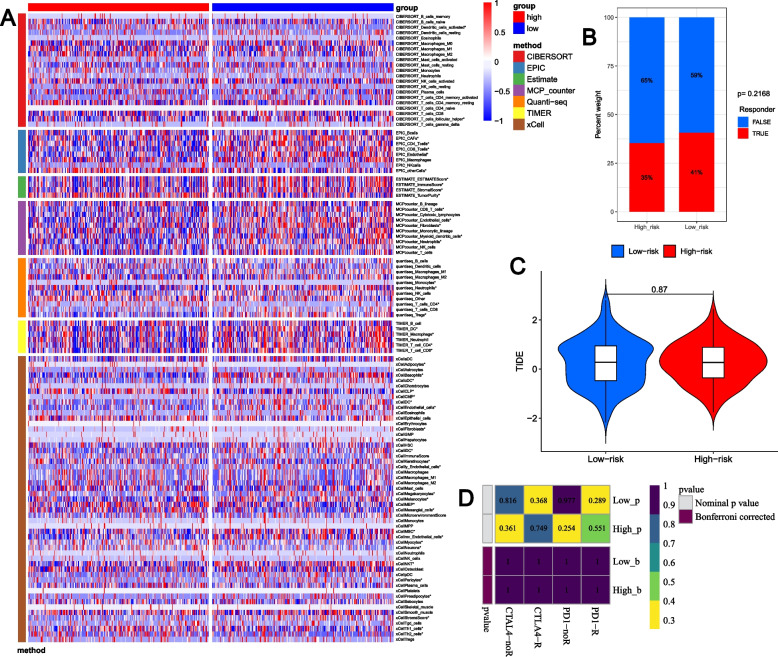
Evaluation of immune activity and immunotherapy for the two GLRG-related risk groups. **A** Analysis of immune activity between the two risk groups using CIBERSORT, EPIC, ESITMATE, MCPcounter, quanTIseq, TIMER and xCell. *P< 0.05. **B** The ICB response rates for the two risk groups. **C** The level of the TIDE scores for the two risk groups. **D** The subclass map showing the immunotherapeutic responses in different risk groups

To further investigate the association between immune activity and GLRG-related signature, we employed computational methods provided by the IOBR R package, including CIBERSORT, EPIC, ESITMATE, MCPcounter, quanTIseq, TIMER, and xCell. Overall, notable disparities were observed in the immune microenvironment between the two risk groups associated with GLRG (Fig. 8A), encompassing dendritic cells activated, T cells follicular helper, CD4 T cells, CAFs, CD8 T cells, Endothelial cells, and so on. However, there was no discernible difference between the two GLRG risk groups’ immunotherapy responses (Fig. 8B-D).

### Drug susceptibility analysis for the two GLRG-related risk groups

**Fig. 9:**
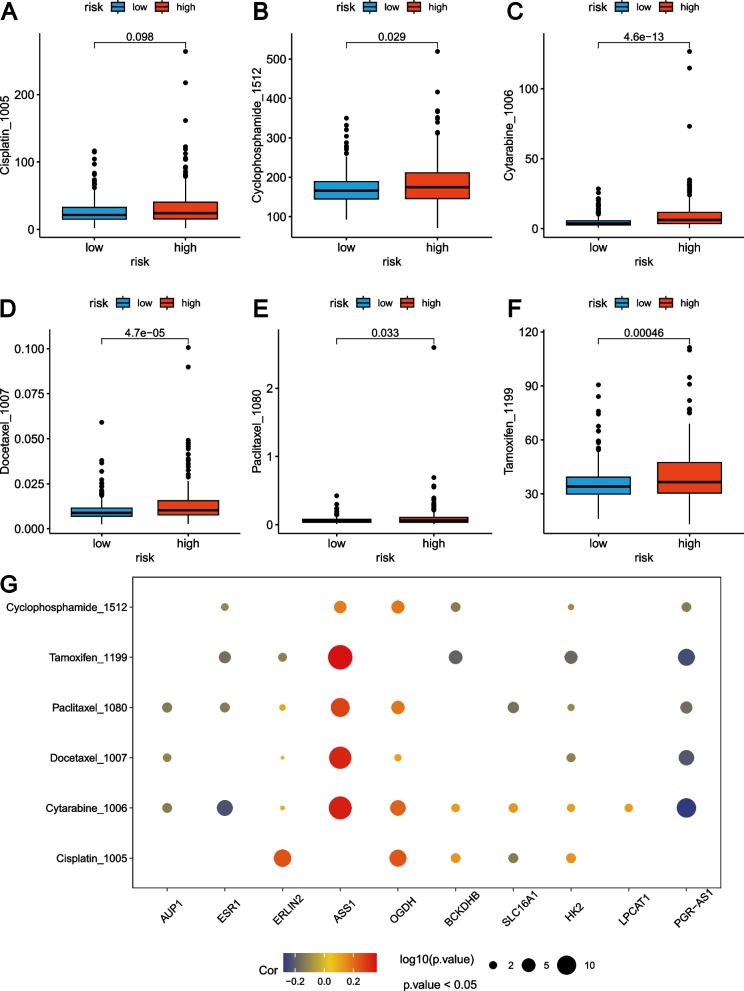
The differences in the chemotherapy response of common chemotherapy drugs between the high-risk group and low-risk group. **A-F** Relationships between risk scores and IC50 level of Cisplatin (**A**), Cyclophosphamide (**B**), Cytarabine (**C**), Docetaxel (**D**), Paclitaxel (**E**), and Tamoxifen (**F**). **G** The Spearman’s correlation coefficients between drug susceptibility and expression levels of the ten genes in the GLRG-related risk model

The drug susceptibility was compared between the two risk groups associated with GLRG. We observed significantly higher IC50 levels of six commonly used chemotherapy drugs for patients in the high-risk group, including Cisplatin (Fig. 9A), Cyclophosphamide (Fig. 9B), Cytarabine (Fig. 9C), Docetaxel (Fig. 9D), Paclitaxel (Fig. 9E), and Tamoxifen (Fig. 9F). These results implied that drug susceptibility and a high risk score are negatively correlated. The drug responsiveness was evaluated in relation to the expression levels of the ten GLRGs employing Spearman’s correlation coefficients (Fig. 9G). Our data demonstrated a positive correlation between the levels of ASS1 and OGDH with multiple drugs, such as Cyclophosphamide, Paclitaxel, Docetaxel, and Cytarabine (Fig. 9G). On the contrary, the levels of AUP1, ESR1, and PGR−AS1 were negatively correlated with several drugs, such as Cytarabine and Paclitaxel(Fig. 9G).

### The expression of GLRGs in the risk model

The expression of the ten GLRGs in EC was examined using quantitative real-time PCR, analyzing ten pairs of clinical samples comprising EC and normal control endometrial tissues. In the TCGA cohort, with the exception of PGR-AS1 (which exhibited lower expression in tumors, the remaining nine GLRGs exhibited significantly higher expression levels in EC tissues (Supplementary Figure S 5). Our findings (Supplementary Figure S 6) were basically consistent with the findings reported in TCGA.

### Knockdown of HK2 suppresses the proliferation, migration, invasion, and promotes the apoptosis of EC cells

**Fig. 10:**
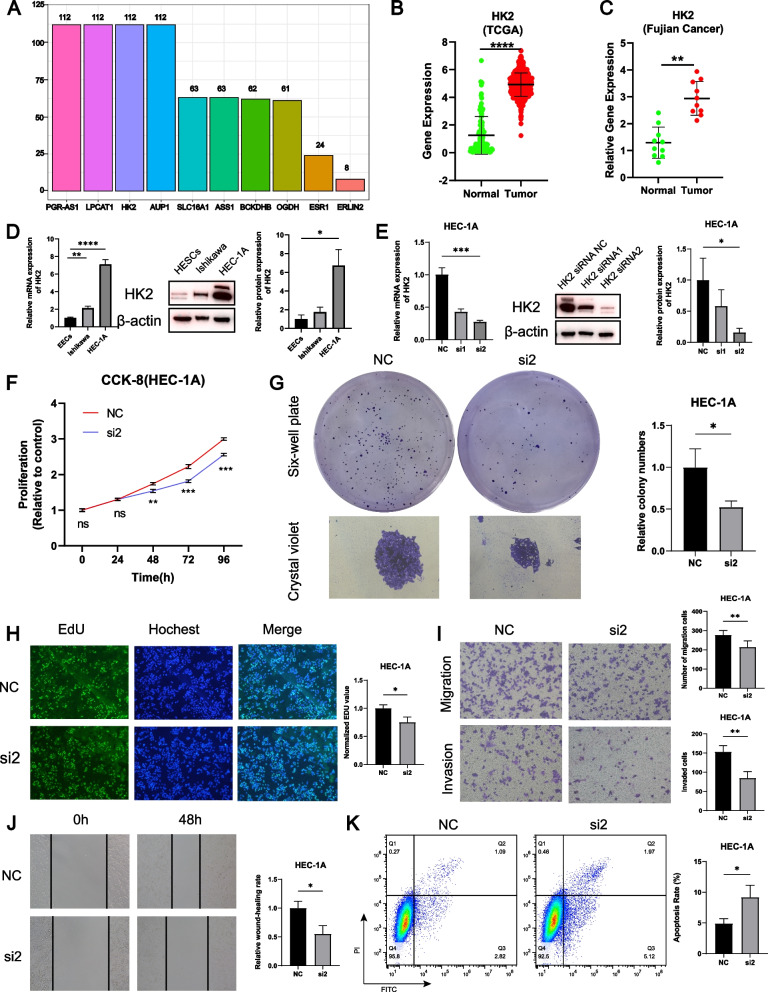
Knockdown of HK2 suppresses the proliferation, migration, invasion, and promotes the apoptosis of EC cells. **A** The frequency of GLRGs observed in the 113 algorithm combinations. **B** The expressed of HK2 in EC based on the data obtained from the TCGA database. **C** The expressed of HK2 in EC based on clinical samples from collected surgical tissue. **D** The expression of HK2 in EECs and EC cells. **E** The knockdown efficiency of HK2 in EC cells. **F** CCK-8 assays of NC and the si2 groups to detect cell viability. The data marked with ns or asterisks were presented as mean ± SD (*n*=3) and subjected to ANOVA analysis. ns: not significant, **P < 0.01, and ***P < 0.001 compared to the NC group at the respective time points (0h, 24h, 48h, 72h and 96h). **G** Colony formation assay. **H** EdU staining were employed to assess cell proliferation. **I** Cell migration and invasion measured through transwell assay. **J** Wound healing assay. **K** Flow cytometry detected cell apoptosis. ns: not significant, *P< 0.05, **P< 0.01, ***P< 0.001, ****P< 0.0001

To further refine the selection of the most significant GLRG among the ten GLRGs in the GLRG-related signature for the subsequent wet experiment, a total of 113 combinations of machine learning algorithms were employed based on the same Train, Test, and All sets. The findings demonstrated that the AUC values of each model exhibited consistently high performance across the Train, Test, and All sets (Supplementary Figure S7). The frequency of GLRGs observed in the 113 algorithm combinations was computed, and we discovered that PGR-AS1, LPCAT1, HK2, and AUP1 exhibited the highest occurrence among the 113 algorithm combinations (Fig. 10A). According to the data obtained from TCGA, we observed a significantly differential expression of HK2 compared to other GLRGs in EC (*P*<0.0001, Fig. 10B, Supplementary Figure S 5). Notably, HK2 exhibited significantly differential expression compared to other GLRGs in the surgically collected tissues (*P*<0.01, Fig. 10C, Supplementary Figure S 6). Hence, we selected HK2 for the follow-up wet experiment. From quantitative real-time PCR (*P*<0.0001) and western blot assays (*P*<0.05), we discovered that HEC-1A cells had considerably higher levels of HK2 expression (Fig. 10D). We transfected HK2-siRNA into HEC1A cells to investigate the functions of HK2 in EC cells. The knockdown efficiency of HK2 was measured, and HK2-siRNA-2(si2) was the sequence with the highest efficiency (*P*<0.001 and* P*<0.05, respectively), therefore, si2 was used in the subsequent functional studies (Fig. 10E). The CCK-8 assays (*P*<0.001, Fig. 10F), colony formation assays (*P*<0.05, Fig. 10G), and EdU assays (*P*<0.05, Fig. 10H) showed that the viability and proliferation of EC cells were inhibited after decreasing the expression level of HK2. The transwell assays (*P*<0.01, Fig. 10I) and scratch wound-healing assays (*P*<0.05, Fig. 10J) revealed a significant reduction in the migration and invasion capacity following HK2 knockdown. In addition, the knockdown of HK2 resulted in a significant increase in apoptosis of EC cells (*P*<0.05, Fig. 10K), as demonstrated by the apoptosis assays. Hence, HK2 is an oncogenic driver that facilitates the progression of EC.

## Discussion

Aberrant glycometabolism and lipid metabolism can exert diverse influences on the development and progression of EC. For instance, elevated blood glucose levels can elicit insulin resistance, a condition characterized by reduced sensitivity of insulin-responsive tissues to insulin, leading to elevated concentrations of both insulin and glucose in the bloodstream. Given that insulin serves as the primary anabolic hormone in the body and exerts its influence on cell proliferation [56], thereby initiating a range of physiological processes associated with carcinogenesis, numerous studies have established a correlation between insulin resistance, incidence of EC, and diverse biological mechanisms [7, 57, 58]. The development of insulin resistance can be induced not only by hyperglycemia but also by obesity, potentially through the promotion of chronic inflammation in adipose tissue and an increase in systemic insulin secretion [59]. Obese individuals exhibit a substantial augmentation in adipose tissue, which actively secretes hormones and adipokines such as leptin and adiponectin. Reduced levels of leptin, elevated circulating adiponectin, and an increased ratio of adiponectin to leptin have been associated with a decreased risk of EC [60]. Another study demonstrated a positive correlation between elevated leptin levels and the progression of EC [61], and cisplatin may potentially exert its therapeutic effects on EC through modulation of the leptin pathway [62]. As previously mentioned, numerous contemporary studies are currently focusing on investigating the impact of aberrant glycometabolism and lipid metabolism on EC. Numerous physiological and pathological processes in vivo exhibit intricate associations with gene expression and its regulation. However, there is currently no established and accurate predictive signature associated with glycometabolism and lipid metabolism-related genes (GLRGs) for prognosticating the outcomes of patients with EC and guiding treatment decisions.

A comprehensive consensus on the risk factors influencing the prognosis of EC patients is yet to be established. In this study, we conducted a comprehensive analysis of the expression profiles of 714 GLRGs in EC using data obtained from the Genecards and TCGA databases. Consequently, we identified differentially expressed GLRGs exhibiting significant prognostic value. The prognostic signature of GLRGs was further established using LASSO Cox regression analysis and subsequently validated for its independent prognostic value. The conventional tumor histology and morphology classification methods fail to capture the full extent of heterogeneity among tumor cells and patients, resulting in poor repeatability and significant variations in prognosis for the same type of tumor. Recent advancements in molecular research on EC have unveiled the genomic alterations associated with its presence, thereby offering valuable insights into the pathogenesis of the disease. Molecular testing holds significant potential in the early detection of EC or precursor lesions and in guiding individualized treatment strategies for EC [63]. The novelty of our study lies in its integration of genes associated with glycometabolism and lipid metabolism, thereby enhancing the precision and generalizability of the predictive model. Based on the GLRG-related signature, patients with EC were stratified into high-risk and low-risk groups. The overall survival (OS) of high-risk EC patients was significantly inferior to that of the low-risk group, even after accounting for clinical factors, as demonstrated across the Train, Test, and Total sets. The accuracy of the risk model in predicting survival status was further validated by consistent findings pertaining to progression-free survival (PFS). The time-varying ROC curve exhibited the resilience of the prognostic GLRG-related signature in accurately predicting survival rates at intervals of 1 year, 3 years, 5 years, 7 years, and 9 years for EC patients across all three sets - Train Set, Test Set, and Total Set. In addition, our study revealed a strong association between higher risk scores, calculated based on the GLRG-related signature, and advanced age, higher stages and grades, as well as an elevated rate of lymph node metastasis overall in patients with EC. The risk score’s C-index and AUC exhibited superior performance in comparison to other clinical features (Age, Grade, Stage, Cluster, and LNM), thereby indicating a stronger predictive power and higher confidence in the GLRG-related signature. Furthermore, the Multivariate Cox regression analysis revealed that the GLRG-related signature exerts an independent influence on the prognosis of patients with EC. Therefore, the GLRG-related signature established in our study facilitates risk assessment, risk stratification, and prognostication for EC patients based on their individual characteristics, thereby equipping clinicians with a valuable tool to evaluate the prognosis of EC and develop appropriate follow-up strategies.

In clinical practice, the implementation of tailored treatment approaches for stratified patients is crucial in enhancing patient prognosis. Tumor immunotherapy represents a therapeutic approach aimed at inducing immune system activation and preventing tumor cells from evading immune surveillance [64]. The challenges of immunotherapy for EC persist in terms of limited response rates, incidence of adverse reactions, and the inability to predict individual efficacy [65]. The immune microenvironment exhibited notable disparities between the two risk groups distinguished by the GLRG-related signature overall. However, no discernible disparity in immunotherapy responses was observed between the two risk groups based on GLRG. Interestingly, the IC50 levels of six commonly employed chemotherapeutic agents were found to be significantly lower in EC patients belonging to the low-risk group, suggesting a heightened sensitivity towards these drugs within this particular subgroup. Among them, Cisplatin exerts inhibitory effects on the DNA replication of EC cells, leading to detrimental consequences on the structural and functional integrity of DNA [62, 66]. Paclitaxel exerts its effects by promoting the inhibition of tubulin polymerization and maintaining tubulin stability, thereby further suppressing tumor cell proliferation [67]. Tamoxifen, a nonsteroidal anti-estrogen drug, exerts its inhibitory effect on the proliferation of EC cells by competitively binding to estrogen receptors and antagonizing estrogen signaling [68]. Regardless of the stage of endometrial cancer, monotherapy has limited efficacy, necessitating adjuvant therapy and combination chemotherapy regimens to enhance treatment outcomes and prognosis. Therefore, our study also holds significant implications for guiding clinical drug utilization.

In order to promote the application of our established risk model, we conducted in vitro experiments on the optimal gene (HK2) within the GLRG-related signature. The rate of glycolysis is primarily regulated by hexokinase, which serves as the initial limiting enzyme in this metabolic pathway. This family of exoglucose-phosphorylase enzymes exhibits a wide distribution across various organisms. The most active isoenzyme within this enzyme family, hexokinase 2 (HK2), plays a pivotal role in glucose metabolism [69]. According to previous studies, HK2 exhibits high expression levels in various malignancies [70], including breast [71], liver [72], colorectal [73], pancreatic [74], and cervical cancers [75]. The heightened glycolytic rate of tumor cells can be attributed to their elevated expression of HK2 [76]. The inhibition of HK2 knockdown not only impeded the tumorigenic growth of glioblastoma, medulloblastoma, and renal cell carcinoma [77–79], but also demonstrated anti-angiogenic effects in pancreatic cancer cells [80]. Numerous inhibitors of HK2 have been developed, including the competitive inhibitor 2-deoxyglucose (2-DG) and the catalytic inhibitors 3-bromopyruvate (3-BrPyr) and clonidamine. In various in vitro and in vivo tumor models, multiple compounds effectively target HK2, inducing its dissociation from mitochondria and subsequently initiating apoptosis in tumor cells [81]. The treatment of type 2 diabetes involves metformin binding to the HK2 and G-6-P sites, resulting in the dissociation of HK2 from mitochondria and facilitating cellular apoptosis. The utilization of metformin as a pivotal preoperative intervention for EC has been extensively employed in the field, effectively restoring atypical endometrial hyperplasia to normal endometrial tissue [82], mitigating the risk of EC, and enhancing the prognosis of patients with EC [83]. Moreover, mitochondrial HK2 governs glycolysis and regulates levels of reactive oxygen species (ROS), while also participating in Ca^2+^ signaling and homeostasis to effectively regulate the energetic survival of cellular organisms [84]. However, the comprehension of HK2 in EC remains limited. Herein, we employed qRT-PCR and WB techniques to validate the robust expression of HK2 in EC tissues. The suppression of HK2 effectively attenuated the proliferation, migration, and invasion of EC cells. The prognostic analysis revealed a significant positive correlation between elevated HK2 expression levels and unfavorable patient outcomes. Our study suggests that HK2 can serve as a reliable biomarker for the diagnosis and prognostic prediction of EC, while targeted regulation of HK2 holds promising potential as a therapeutic intervention for managing EC.

Although there have been published articles specifically examining the prognostic characteristics of EC [85], their focus has been exclusively limited to one aspect of metabolism. The present study constitutes the initial endeavor to integrate genes linked with glycometabolism and lipid metabolism. Nevertheless, it is necessary to delineate the existing limitations within this study. The accuracy of the GLRG-related risk model in predicting prognosis has been validated using the TCGA database; however, further collection of EC samples, external independent datasets, and extensive prospective clinical analysis are necessary to validate the effectiveness and utility of the GLRG-related signature and biomarkers in clinical applications. The correlation between the GLRG-signature and drug susceptibility necessitates further validation through clinical trials and molecular biology experiments. Other clinical factors, such as lymphovascular space invasion (LVSI), have been identified as significant prognostic indicators in EC. However, due to limited data availability, the inclusion of LVSI in this study is currently unfeasible. Additionally, the underlying mechanism through which HK2 facilitates EC progression remains elusive, and our study has solely undergone experimental validation in vitro. A comprehensive understanding of the potential mechanism of HK2 necessitates extensive exploration and experimentation in vivo.

### Supplementary Information

**Supplementary Material 1.**

**Supplementary Material 2.**

**Supplementary Material 3.**

**Supplementary Material 4.**

**Supplementary Material 5.**

**Supplementary Material 6.**

**Supplementary Material 7.**

## Data Availability

The RNA sequencing profiles are able to be gained from The Cancer Genome Atlas (TCGA) (https://toil.xenahubs.net) and Gene Expression Omnibus (GEO) database (https://www.ncbi.nlm.nih.gov/geo/). Further inquiries can be directed to the corresponding author.
