# Supplementary material for: Glycometabolism and lipid metabolism related genes predict the prognosis of endometrial carcinoma and their effects on tumor cells
Source: BMC Cancer. 2024 May 8;24:571. doi: 10.1186/s12885-024-12327-1 (PMC11080313; doi:10.1186/s12885-024-12327-1)
Supplement: Supplementary file 1 — Supplementary Material 1. [file 12885_2024_12327_MOESM1_ESM.docx]

**Glycometabolism and lipid metabolism related genes predict the prognosis of endometrial carcinoma and their effects on tumor cells**

**Xuefen Lin^1†^, Jianfeng Zheng^1†^, Xintong Cai^1^, Li Liu^1^, Shan Jiang^1^, Qinying Lin^2^, Yang Sun^1, *^**

^1^Department of Gynecology, Clinical Oncology School of Fujian Medical University, Fujian Cancer Hospital, Fuzhou, 350014, China

^2^Fujian provincial key laboratory of tumor biotherapy, Fujian Cancer Hospital, Fuzhou, 350014, China

*** Correspondence:**

Correspondence should be addressed to Yang Sun.

Address: No.420, Fuma Road, Jin ‘an District, Fuzhou City, Fujian Province, P. R. China

Email: [sunyang@fjzlhospital.com](mailto:sunyang@fjzlhospital.com)

**^†^** Xuefen Lin and Jianfeng Zheng contributed equally to this work.


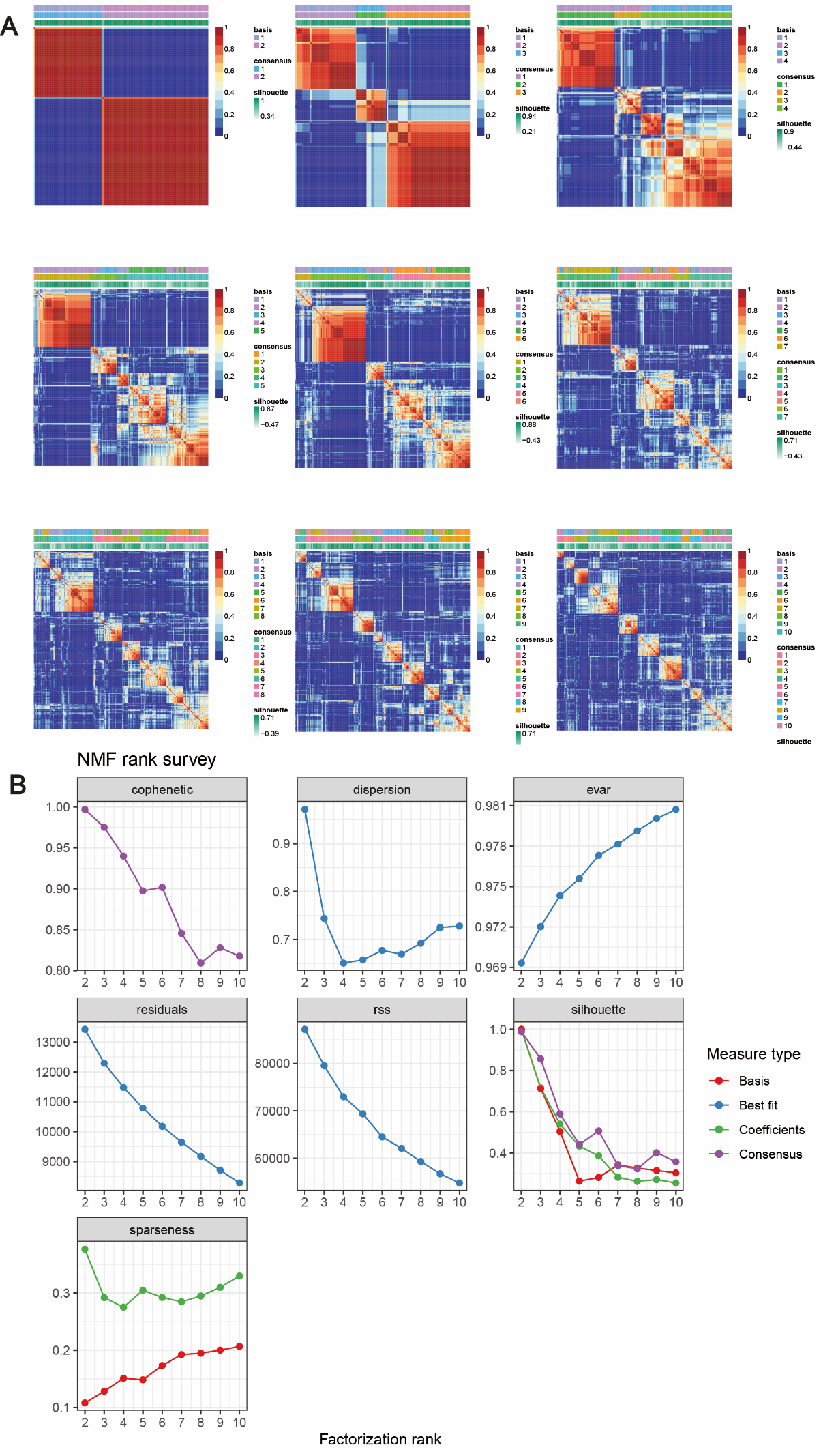


**Supplementary Figure S1.** NMF clustering consensus maps. **(A)** Heatmap showing the differentially expressed Glycometabolism and lipid metabolism related genes in TCGA. **(B)** NMF clustering consensus map.


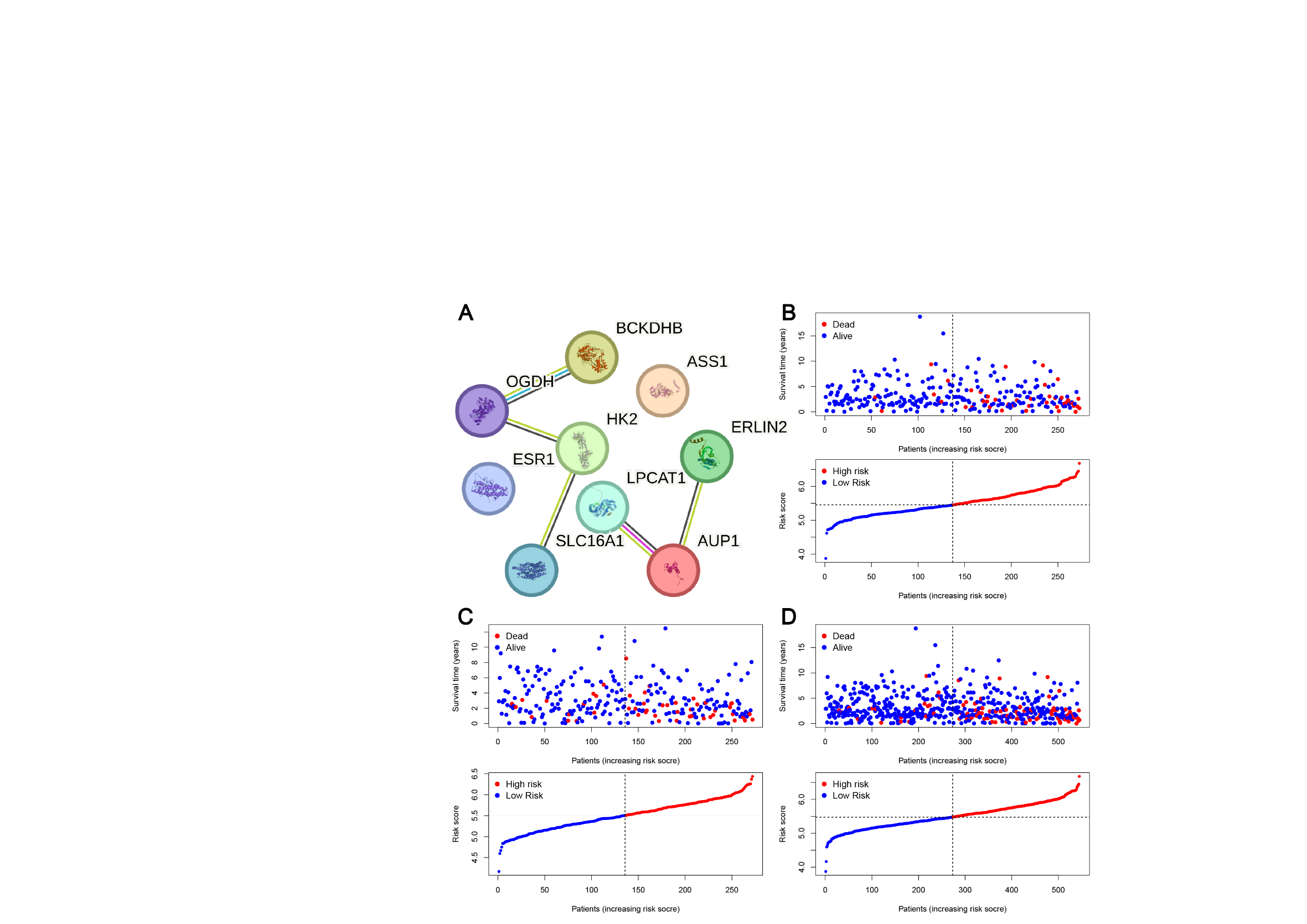


**Supplementary Figure S2.** **(A)** Protein–protein interaction network of 10 GLRGs. Risk scores distribution and survival status of EC patients in Train Set **(B)**, Test Set **(C)** and Total Set **(D)**.


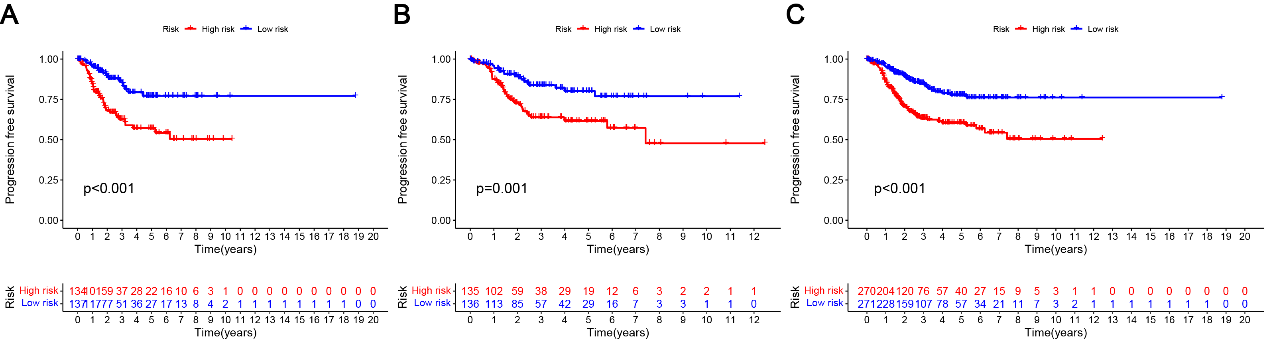


**Supplementary Figure S3.** The progression free survival (PFS) for patients in Train Set **(A)**, Test Set **(B)** and Total Set **(C)**.


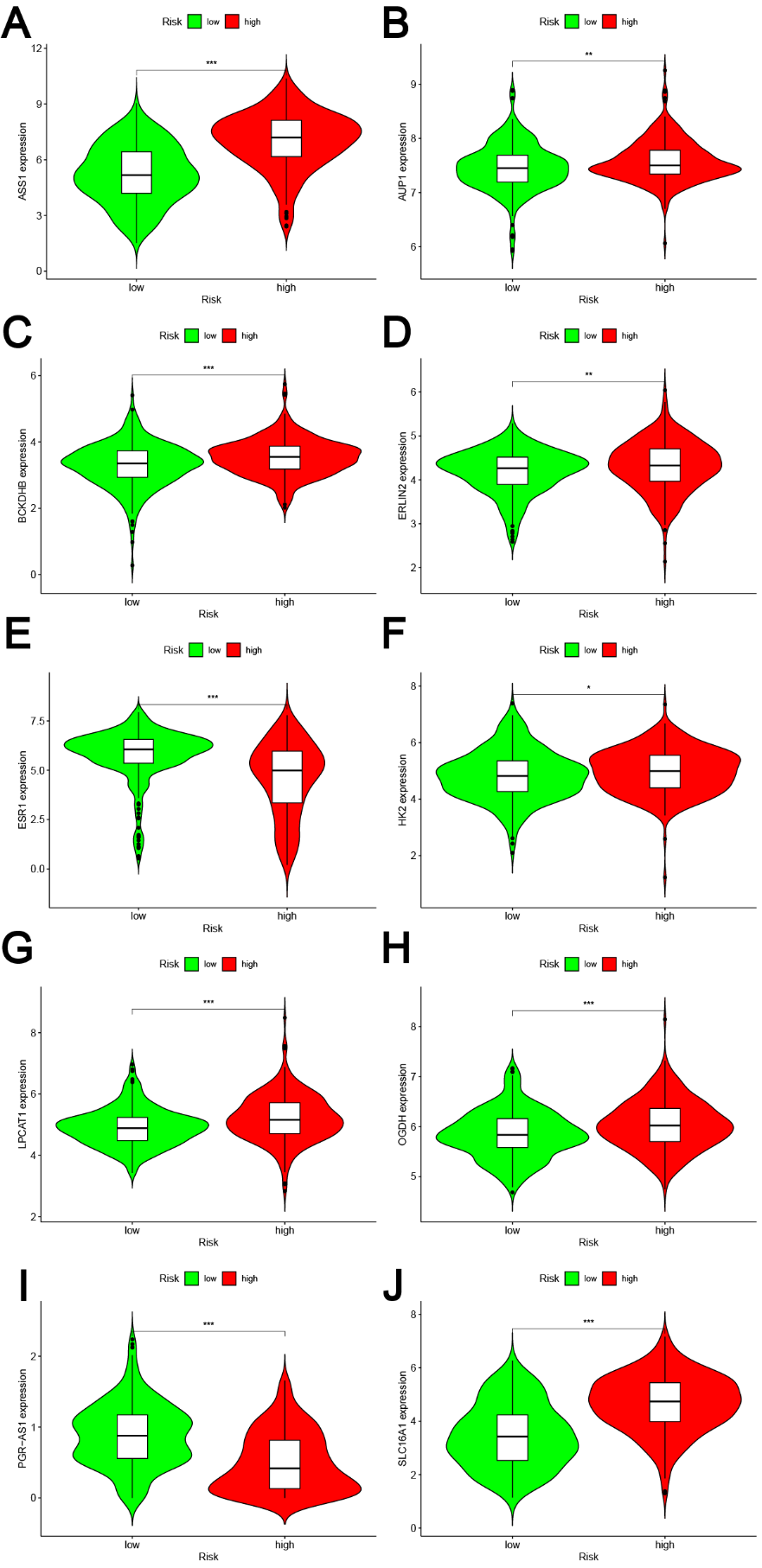


**Supplementary Figure S4.** The expression levels of the ten GLRGs between low-risk group and high-risk group. **(A)** ASS1, **(B)** AUP1, **(C)** BCKDHB, **(D)** ERLIN2, **(E)** ESR1, **(F)** HK2, **(G)** LPCAT1, **(H)** OGDH, **(I)** PGR−AS1, **(J)** SLC16A1.


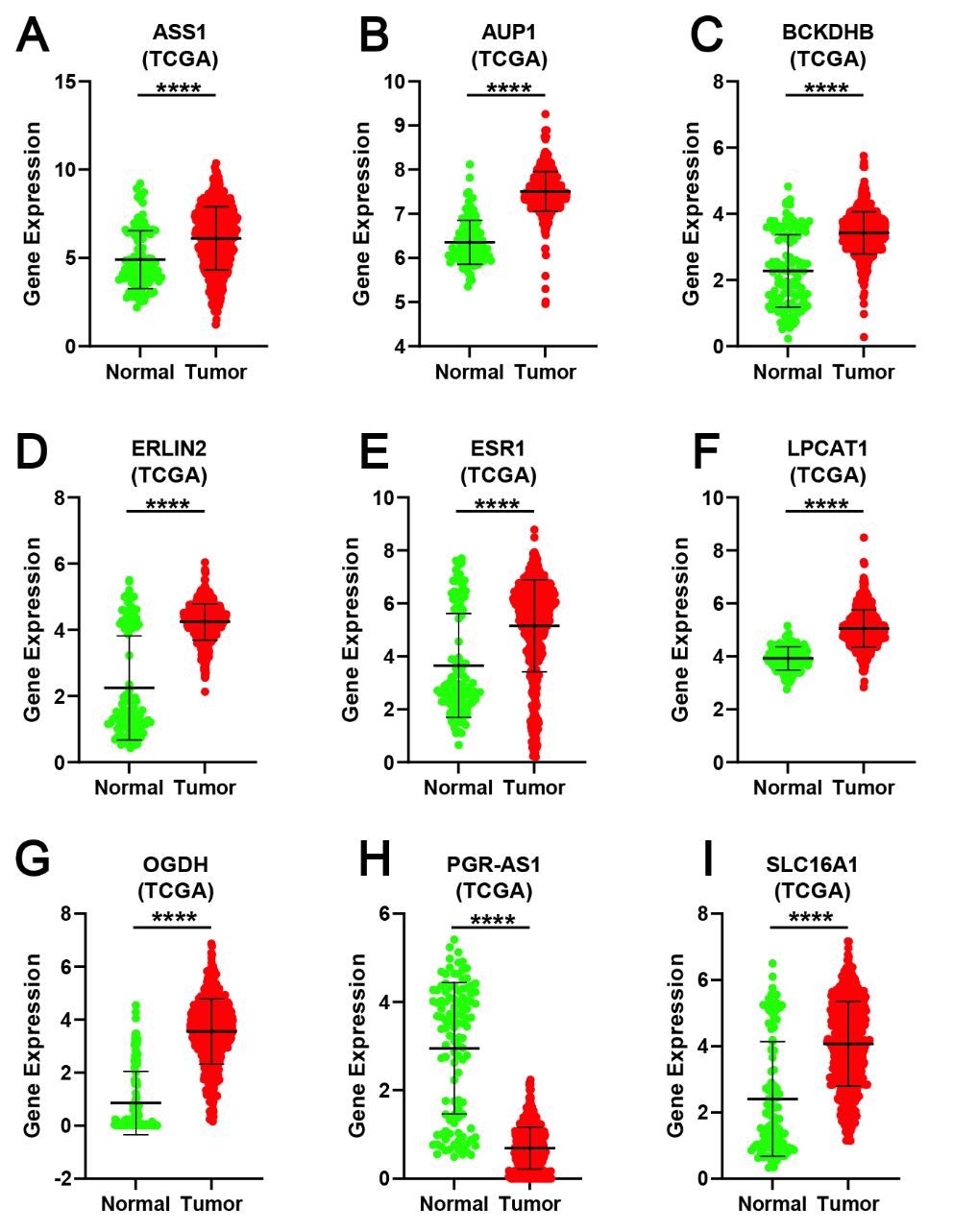


**Supplementary Figure S5.** The expression of the GLRGs in TCGA database. **(A)** ASS1, **(B)** AUP1, **(C)** BCKDHB, **(D)** ERLIN2, **(E)** ESR1, **(F)** LPCAT1, **(G)** OGDH, **(H)** PGR−AS1, **(I)** SLC16A1.


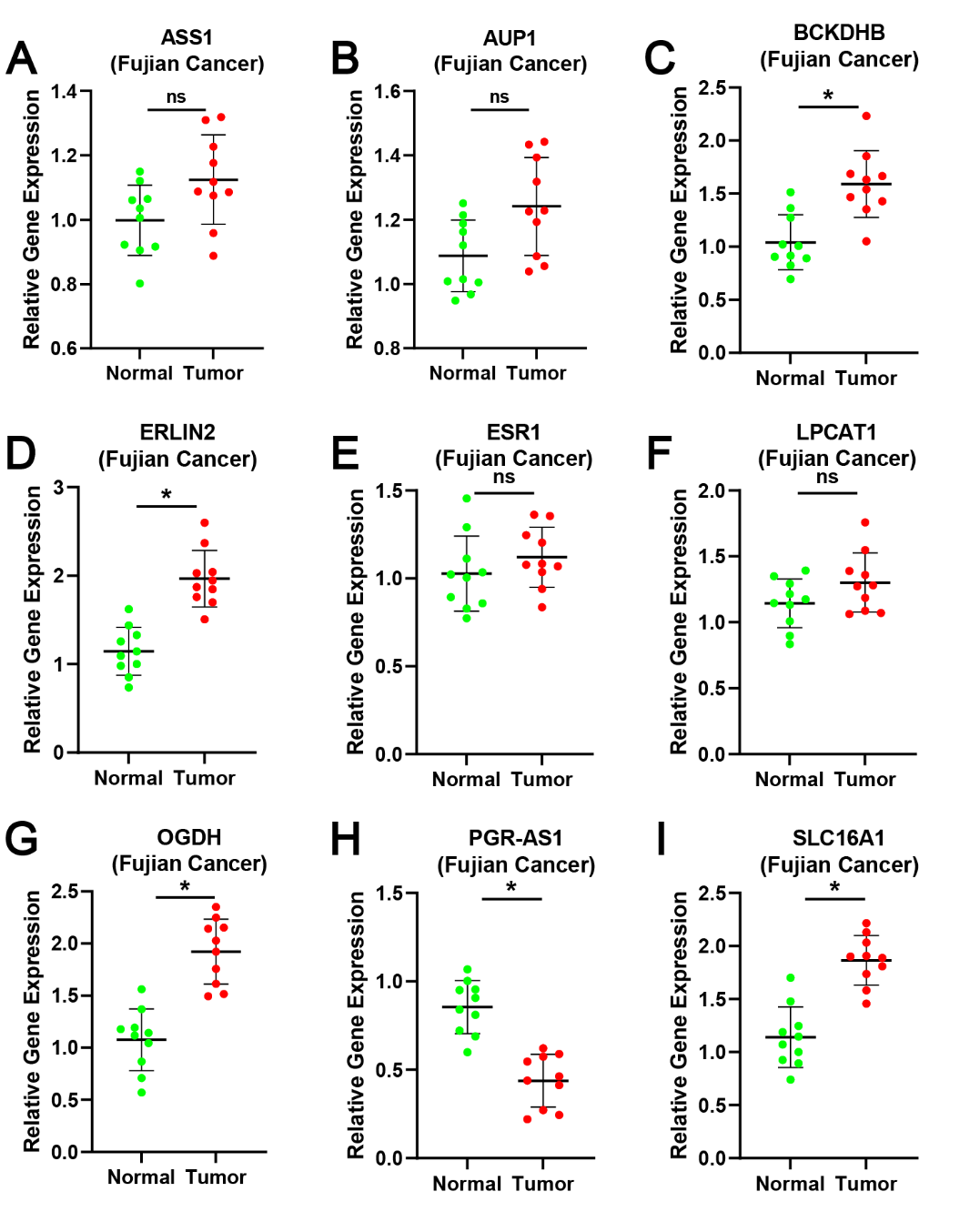


**Supplementary Figure S6.** The expression of the GLRGs in collected surgical tissue. **(A)** ASS1, **(B)** AUP1, **(C)** BCKDHB, **(D)** ERLIN2, **(E)** ESR1, **(F)** LPCAT1, **(G)** OGDH, **(H)** PGR−AS1, **(I)** SLC16A1.


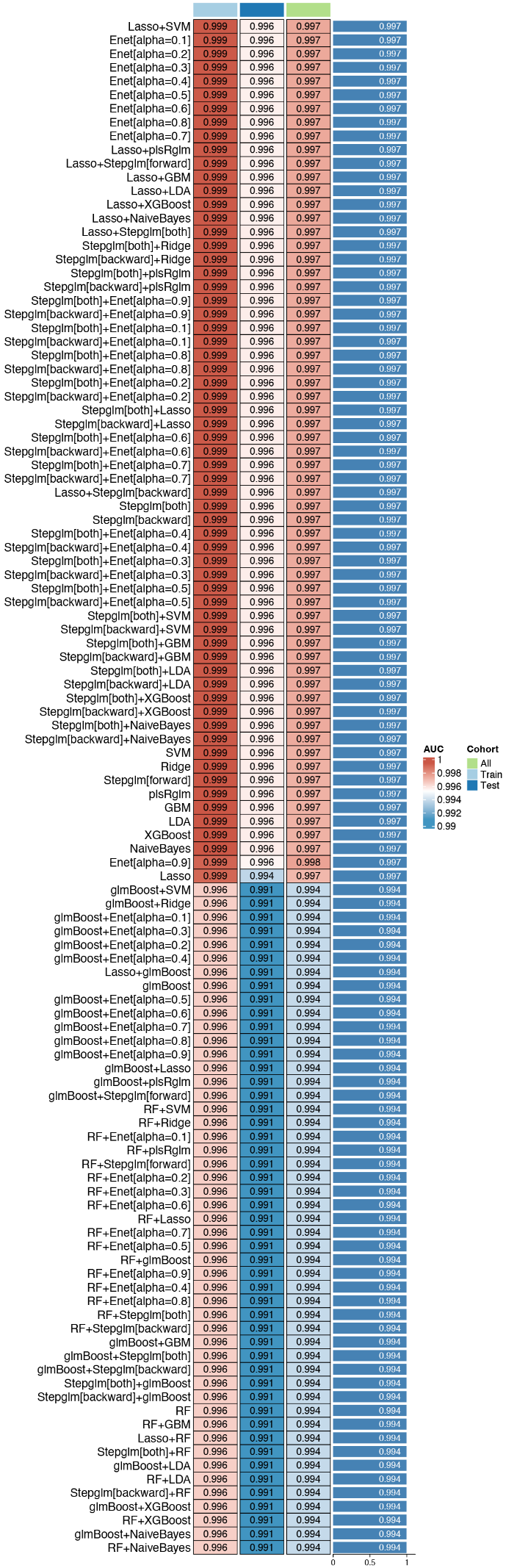


**Supplementary Figure S7.** The AUC of each model was calculated using 113 ML algorithm. The 113 algorithms were based on the calculation results of the same training set and test set.
