# Supplementary figures and images for: Glycometabolism and lipid metabolism related genes predict the prognosis of endometrial carcinoma and their effects on tumor cells
Source: BMC Cancer. 2024 May 8;24:571. doi: 10.1186/s12885-024-12327-1 (PMC11080313; doi:10.1186/s12885-024-12327-1)

**Figure 10D-E**


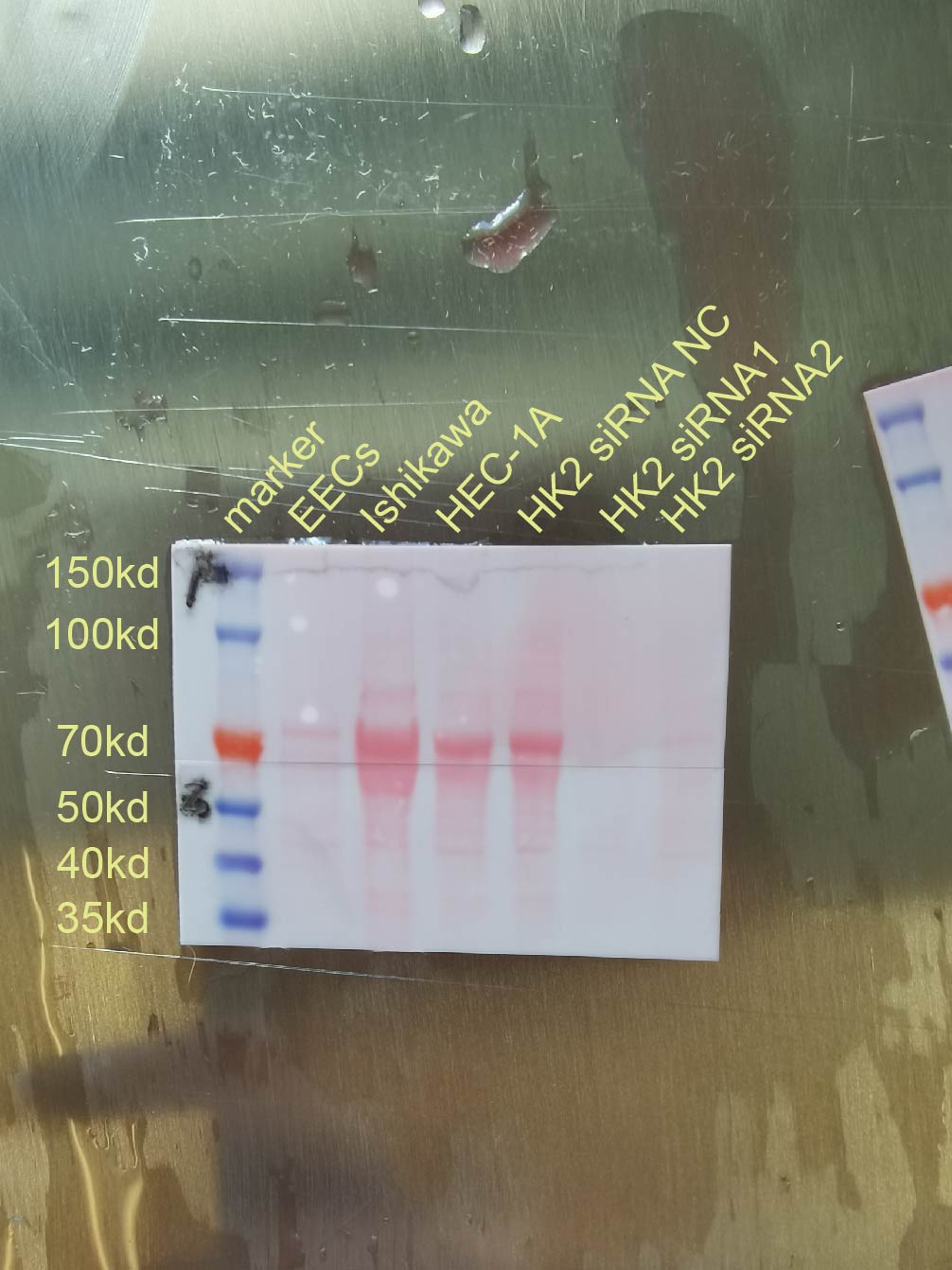


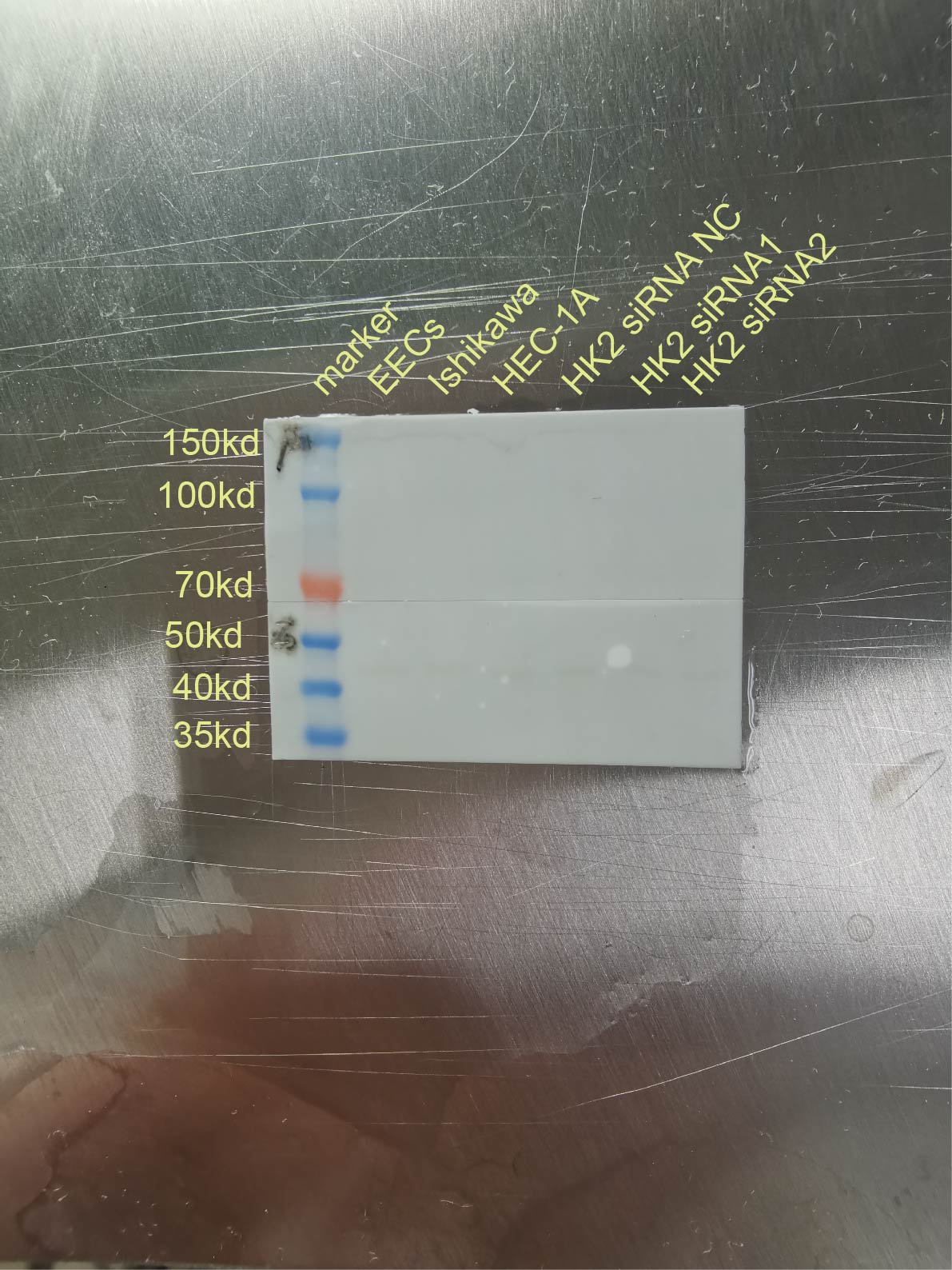


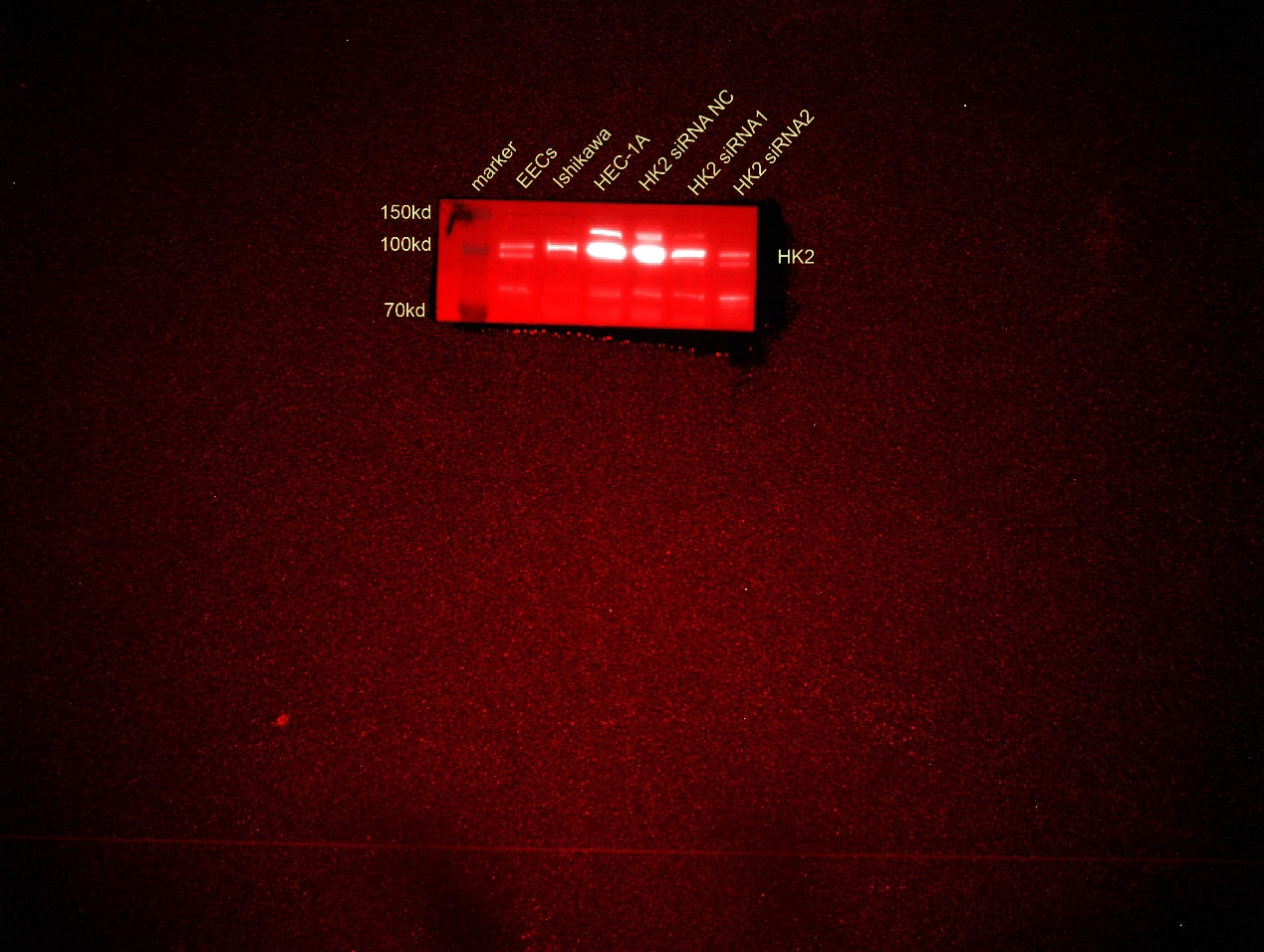


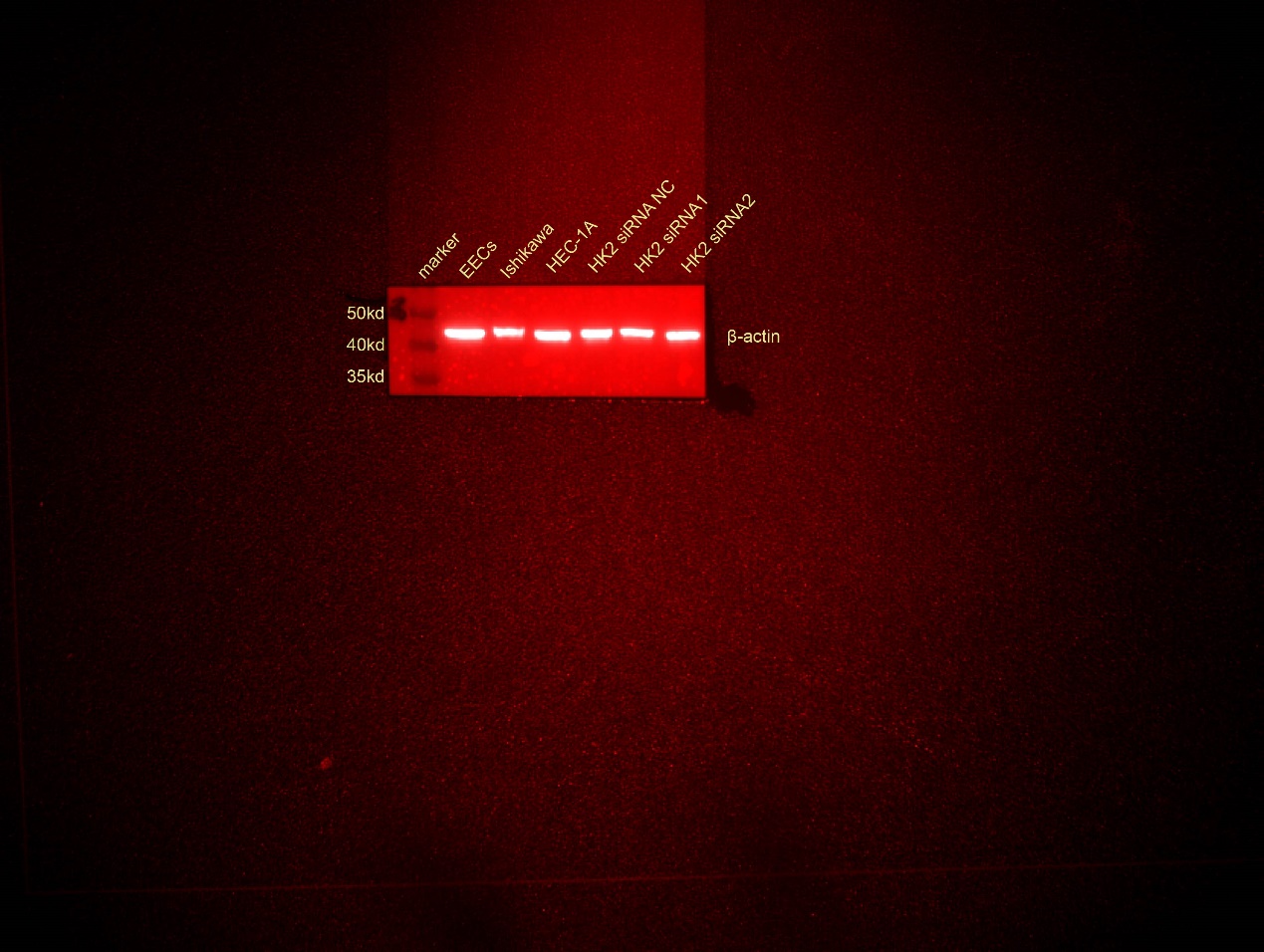


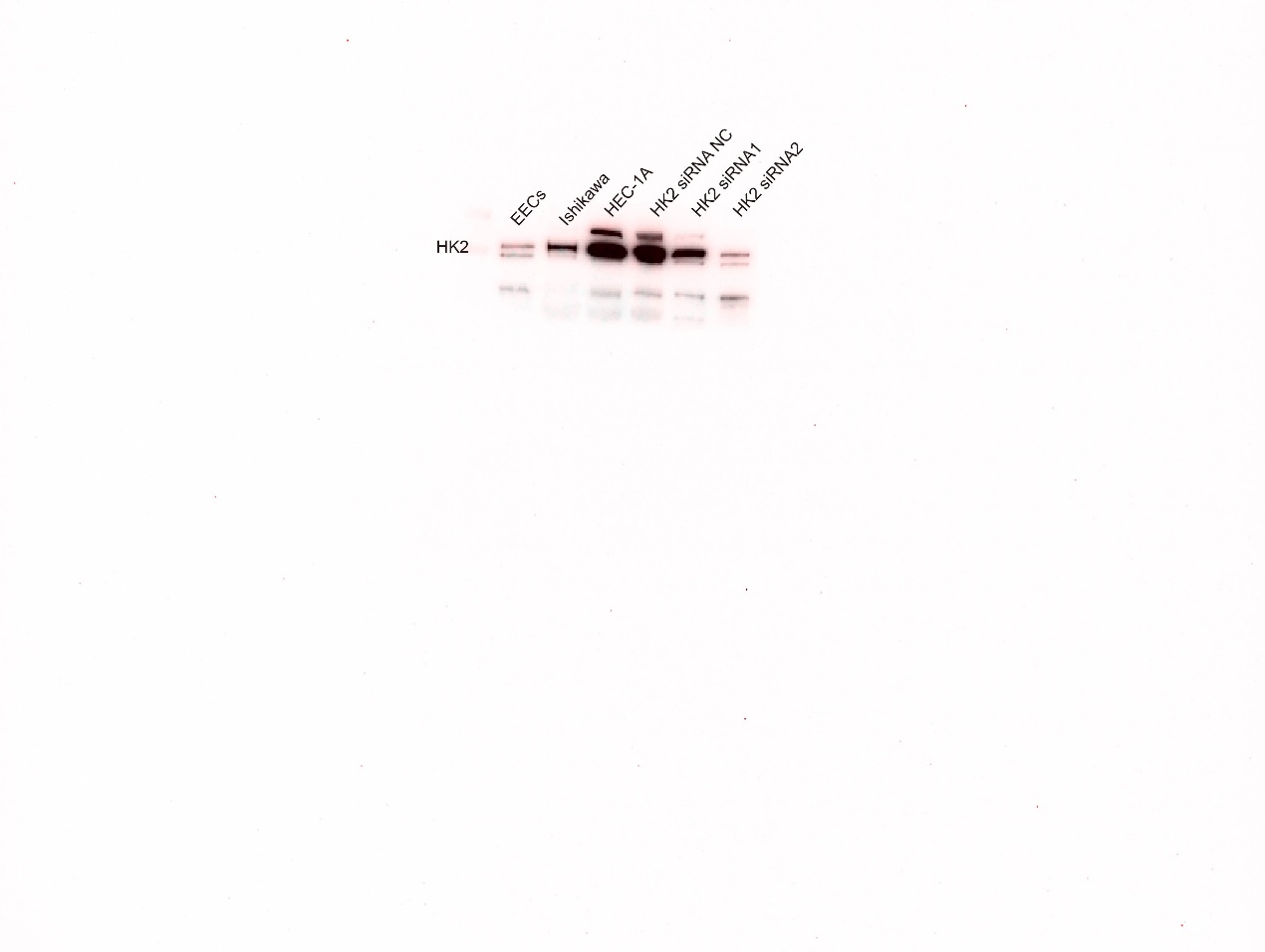


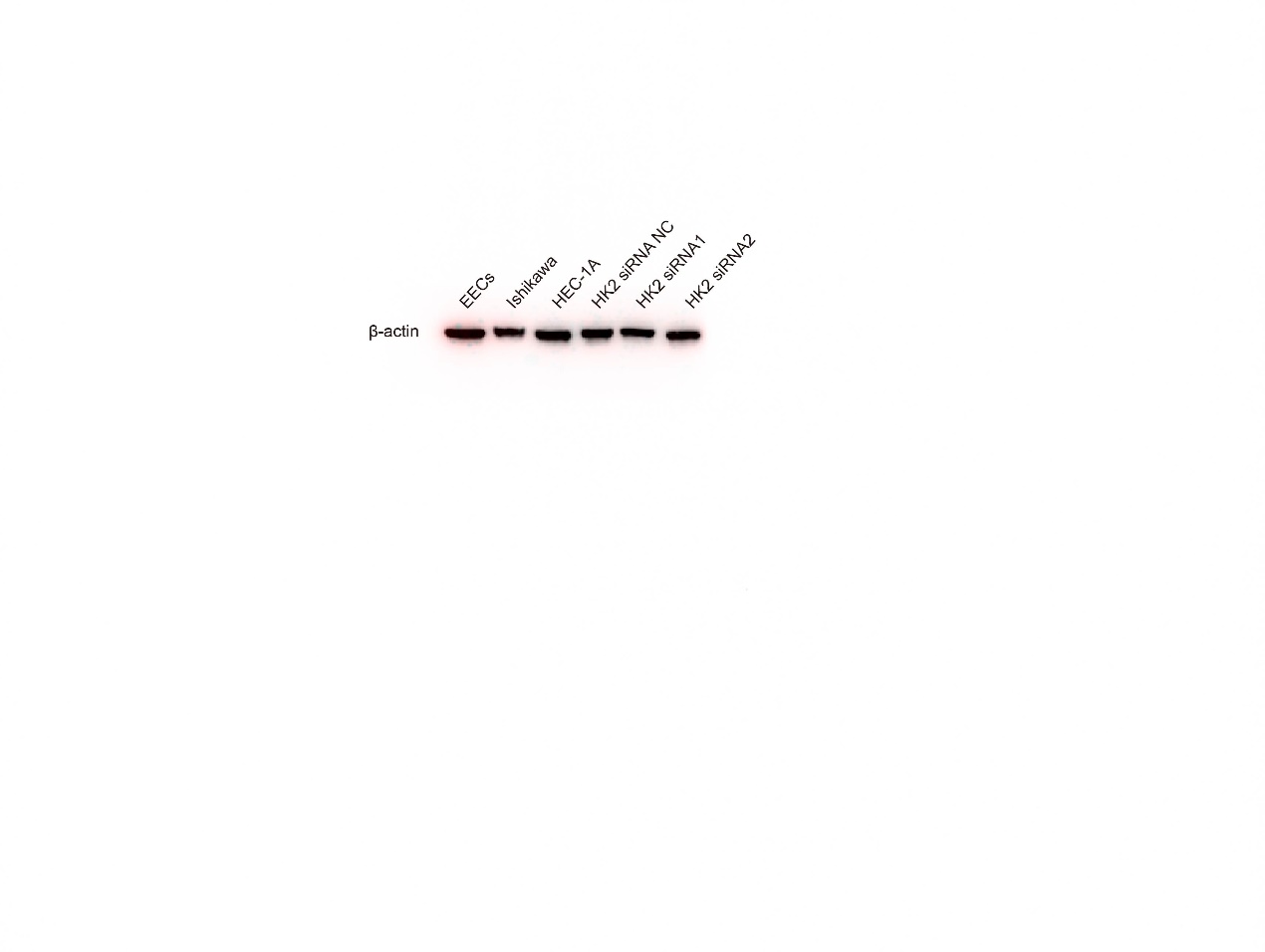

Supplement: Supplementary file 7 — Supplementary Material 7. [file 12885_2024_12327_MOESM7_ESM.docx]
